# Collagen scaffold enhances the regenerative properties of mesenchymal stromal cells

**DOI:** 10.1371/journal.pone.0187348

**Published:** 2017-10-31

**Authors:** Iran Rashedi, Nilesh Talele, Xing-Hua Wang, Boris Hinz, Milica Radisic, Armand Keating

**Affiliations:** 1 Institute of Biomaterials and Biomedical Engineering, University of Toronto, Toronto, Canada; 2 Cell Therapy Program, University Health Network, Toronto, Canada; 3 Laboratory of Tissue Repair and Regeneration, Matrix Dynamics Group, Faculty of Dentistry, University of Toronto, Toronto, Canada; 4 Arthritis Program, Krembil Research Institute, University Health Network, Toronto, Canada; 5 Princess Margaret Cancer Centre, University Health Network, Toronto, Canada; Michigan State University, UNITED STATES

## Abstract

MSCs are widely applied to regenerate heart tissue in myocardial diseases but when grown in standard two-dimensional (2D) cultures exhibit limited potential for cardiac repair and develop fibrogenic features with increasing culture time. MSCs can undergo partial cardiomyogenic differentiation, which improves their cardiac repair capacity. When applied to collagen patches they may improve cardiac tissue regeneration but the mechanisms remain elusive. Here, we investigated the regenerative properties of MSCs grown in a collagen scaffold as a three-dimensional (3D) culture system, and performed functional analysis using an engineered heart tissue (EHT) model. We showed that the expression of cardiomyocyte-specific proteins by MSCs co-cultured with rat neonatal cardiomyocytes was increased in collagen patches versus conventional cultures. MSCs in 3D collagen patches were less fibrogenic, secreted more cardiotrophic factors, retained anti-apoptotic and immunomodulatory function, and responded less to TLR4 ligand lipopolysaccharide (LPS) stimulation. EHT analysis showed no effects by MSCs on cardiomyocyte function, whereas control dermal fibroblasts abrogated the beating of cardiac tissue constructs. We conclude that 3D collagen scaffold improves the cardioprotective effects of MSCs by enhancing the production of trophic factors and modifying their immune modulatory and fibrogenic phenotype. The improvement in myocardial function by MSCs after acquisition of a partial cardiac cell-like phenotype is not due to enhanced MSC contractility. A better understanding of the mechanisms of MSC-mediated tissue repair will help to further enhance the therapeutic potency of MSCs.

## Introduction

MSCs continue to be investigated for the restoration of myocardial function after injury in preclinical and clinical settings. Conventional monolayer cultures on two-dimensional (2D) plastic surfaces, however, poorly represent the *in vivo* microenvironment. Various three-dimensional (3D) cell cultures have been developed to mimic the extracellular microenvironment in which cells naturally reside, and are used to model solid tissues and provide a platform for cell growth and transport. 3D collagen substrates are an attractive bioengineering approach to repair myocardium, because collagen is a natural polymer and major constituent of the myocardium extracellular matrix (ECM) [1,2]. Collagen patches seeded with bone marrow (BM)-derived MSCs have been evaluated and proven clinically safe [3]. Although the therapeutic benefits of MSCs delivered with such patches have been mainly attributed to enhanced cellular retention at the site of tissue injury, 3D substrates also alter biological properties of MSCs, including lineage differentiation capacity [4–10] and enhance their therapeutic potency [8,11,12]. The mechanism(s) of this functional improvement however are largely unknown.

We and others have shown that MSCs acquire a cardiomyocyte-like phenotype in cardiomyogenic environments such as in co-culture with primary cardiomyocytes. These MSC-derived cardiomyocyte-like cells show increased expression of several cardiomyocyte-specific markers but lack the sarcomeric organization and ionic currents and do not generate action potentials characteristic of functional cardiomyocytes [13–16]. This acquisition of a cardiac cell-like phenotype, although partial, improves the ability of MSCs to restore myocardial function in animal models [17–21]. Based on the results of these studies, a clinical trial has been initiated using autologous MSCs primed with a cardiogenic cocktail in patients with chronic heart failure and history of ischemic disease [22]. The ability of MSCs to acquire a partial cardiac cell phenotype is enhanced in various 3D models. Nevertheless, the mechanism(s) of the functional improvement in MSCs that adopt a partial cardiomyocytic phenotype remain elusive. A better understanding of how MSCs promote cardiac tissue repair is likely to lead to enhanced functional potency and to improve therapy for these challenging disorders. In this study, we looked at the properties of human BM-MSCs important for regenerative function in 2D and 3D cultures, and examined their electromechanical function in an engineered heart tissue model (EHT).

## Materials and methods

### Cell isolation, characterization and culture

#### MSC and human dermal fibroblast

Bone marrow and blood samples were obtained from healthy volunteers after informed written consent as approved by the University Health Network Research Ethics Board (protocol numbers 06-446-CE and 15-9224-CE, respectively). MSCs were isolated from bone marrow aspirates by plastic adherence. Briefly, erythrocytes were lysed in Ca^2+^/Mg^2+^-free phosphate buffered saline (PBS) containing 2mM EDTA for 5 min at room temperature (RT). The mononuclear cell fraction was separated by the Ficoll-Paque (GE Healthcare Life Sciences, Mississauga, ON) gradient centrifugation method. Cells were washed in PBS and suspended in Dulbecco’s Modified Eagle’s Medium (DMEM/ 1 g/L glucose, Life Technologies, Burlington, ON) supplemented with 10% fetal bovine serum (FBS), 100 U/mL of penicillin and 100 μg/mL of streptomycin (Life Technologies). Cells were plated at 5 × 10^7^ cells/175 cm^2^ in tissue culture flasks and maintained at 37°C in a humidified incubator containing 5% CO_2_. MSCs were characterized by the expression of CD73, CD105, CD90 and lack of the expression of hematopoietic markers CD11b, CD14, CD19, CD34, CD45, and HLA-DR2 on their surface (as described in flow cytometry analysis section) as well as their tri-lineage differentiation capacity using a commercially available kit (StemPro, TermoFisher Scientific) following manufacturer’s instructions. MSCs at passage 3–6 were used for the experiments and cultured at a density of 1.5×10^4^ cells/cm^2^. Human dermal fibroblasts (ATCC^®^, PCS-201-012) were cultured under similar conditions as MSCs.

#### Lymphocyte and monocyte

Peripheral blood mononuclear cells (PBMCs) were isolated by the Ficoll-Paque density gradient method. Monocytes were isolated from PBMCs by positive selection using magnetic activated cell sorting (MACS) and anti-CD14 microbeads according to manufacturer’s instructions (Miltenyi Biotech, San Diego, CA). CD14(-ve) fractions were enriched for CD4(+) T lymphocytes by negative selection using a kit (Stem Cell Technologies, Vancouver, BC), and maintained in RPMI supplemented with 10% FBS, 1% sodium pyruvate and 1% penicillin/streptomycin.

#### Neonatal rat cardiomyocytes

Neonatal rat cardiomyocytes (rCMs) were isolated from 1 or 2-day-old rats (Sprague–Dawley, Charles River Laboratories) according to a protocol approved by the University of Toronto Animal Care Committee (protocol number 20011246). Whole hearts were cut and enzymatically digested using trypsin (6120 U/mL, 4°C, 5–8 h, Sigma-Aldrich, Oakville, ON), followed by serial collagenase digestions (37°C, 5 steps, 8 min each, Worthington Biochemical, Lakewood, NJ). Cell suspensions were enriched for rCMs by centrifugation and two rounds of pre-plating (37°C, 1 h each). The purity of cardiomyocytes (i.e. the ratio of cardiomyocytes to cardiac fibroblasts/cFb) was assessed by flow cytometery using antibodies for cardiac proteins as described in “flow cytometery analysis” section. Isolates containing more than 70% cardiac marker-expressing cells were used for downstream experiments. Cells were cultured on plates coated with 0.7% gelatin (Anachemia, cat. 4622) at 1.5×10^5^/cm^2^ in DMEM/F12 (Life Technologies) supplemented with 10% horse serum (Invitrogen, cat. 16050122), 1% FBS, and 1% penicillin/streptomycin. For fibrosis-associated studies, culture plates were coated with 10 μg/cm^2^ collagen (type I, Bovine, Life Technologies) overnight at 4°C. Excessive solution was removed, plates left to air dry, and rinsed thoroughly before cell culture.

### 3D cultures

Custom-sized patches (1 cm × 0.5 cm × 300 μm) were prepared from a commercially-available type-I bovine collagen substrate (Ultrafoam, Davol, Warwick, RI). Patches were UV-sterilized and soaked in culture medium before cell culture. For seeding the cells on and into patches, MSCs (5×10^4^) or rCM/cFb (5×10^5^) were mixed with Matrigel (5 μl, BD Bioscience, San Jose, CA) which facilitated the uniform spread of cells into the patch. In order to obtain similar cell densities between 2D and 3D cultures, the 300 μm thickness of each patch was considered to accommodate approximately six stacks of cell layers (average MSC and CM cell size of 10–100μm), based on which the number of cells cultured per surface area in plates were multiplied by a factor of six and equivalent numbers of cells were cultivated into each patch. Patches were allowed to gel for 20 min in a humidified incubator at 37°C before culture medium was added to the wells in which patches were floating. Fresh media was supplemented every two days. Cells were maintained in patches for 3 days to allow establishing new phenotype before being used in downstream experiments. MSCs cultivated in collagen patches were subsequently used in downstream experiments as 3D-MSCs, and rCM/cFb-containing patches were either used for co-culture with MSCs or utilized as cardiac tissue constructs for electro-functional analyses.

### Cell stimulation

An inflammatory environment was simulated by incubating MSCs with Poly (I:C) (TLR3 agonist, 20 μg/mL for 6 h, Sigma-Aldrich) or LPS (TLR4 agonist, 10 ng/mL, 1 h, Sigma-Aldrich). To induce a pro-fibrotic phenotype, cells were incubated with recombinant human TGF-β1 (5 ng/mL for 48 h, Life Technologies) for 48 h. Cells treated with recombinant human IFN-γ (interferon-gamma, 500 U/mL for 24 h, US Biological Salem, MA) were used as the negative control. Cells were washed a minimum of three times to remove residual stimulating agents before being used in subsequent experiments.

### Co-culture assays

Schematic experimental design of MSC mono- and co-cultures are depicted in S1 Fig.

#### Co-culture with rCM/cFb

On day 2, when cultured cardiomyocytes started to beat (monolayer or entire patch), MSCs were added to rCM/cFb at a ratio of 1:10 (MSC:rCM/cFb). For adding cells into the collagen patches, similar protocol, as described in 3D culture, was followed (i.e. cells mixed with matrigel were spot-seeded in the patches and allowed for gel formation in the incubator before the culture medium was added to the wells). Co-cultured cells were incubated for another 3 to 5 days (for gene expression and for being further used in functional analyses, respectively) in MSC- and rCM-specific growth media (1:1 ratio). When applicable, hdFbs were co-cultured with rCM/cFb in a similar fashion and used as control to evaluate cell-specific effect(s).

#### Co-culture with lymphocytes

To assess the immunosuppressive function in 2D and 3D cultures, MSCs were co-cultured with allogeneic CD4(+) lymphocytes. MSCs (1×10^4^) were cultured as monolayers in 96-well plates one day before CD4(+) T cells labeled with carboxyfluorescein succinimidyl ester (CFSE, Life Technologies) and activated with CD3/CD28 Dynabeads (bead to cell ratio of 1:20, Life Technologies) were added to the wells (1:10 MSC:lymphocyte). In 3D cultures, MSCs were cultured in collagen patches (0.3 cm×0.3 cm×300 μm or 0.5 cm×0.5 cm×300 μm) for 3 days before patches were transferred to freshly-cultured lymphocytes in 96-well plates. Cells were maintained in co-culture for another 4 days before analysis by flow cytometry. Mono-cultured lymphocytes were used as control.

#### Co-culture with monocytes

To study macrophage polarization, MSCs (2.5×10^4^) were cultured on 24-well plates before CD14(+) monocytes activated toward M1 phenotype with human granulocyte macrophage colony-stimulating factor (GM-CSF, 10 ng/mL, PeproTech, Rocky Hill, NJ) were added to the wells (1:3 MSC:monocyte). In 3D cultures, MSCs cultured in collagen patches (0.3 cm×0.3 cm×300 μm or 0.5 cm×0.5 cm×300 μm) for 3 days were transferred to freshly-cultured monocytes in 24-well plates. Identical 2D and 3D MSC cultures were prepared for the generation of MSC-conditioned media for corresponding indirect co-culture experiments. Cells were incubated for 4 more days before flow cytometry analysis. Mono-cultured monocytes were used as control. For experiments with conditioned media, the MSC-conditioned medium was collected from 2D and 3D MSC cultures in 24-well plates. The cell-free medium was concentrated using 3K Centrifugal Filters (Millipore, Etobicoke, ON) according to the manufacturer’s instructions. The fold concentration varied within a range of 8–11 fold (36–50 μL) for each well. The entire concentrated medium was mixed with fresh medium to a final volume similar to all other experimental groups (400 μL) and added to monocytes (7.5×10^4^) cultured in 24-well plates.

### Electro-functional analysis of tissue constructs

EHT was used for functional analysis as previously described [23]. We seeded rCM/cFb (5×10^5^) in the collagen patches but did not subject them to electrical stimulation during the culture period. When the patches began to synchronously beat on day 2, equal numbers (1×10^5^) of cells from each experimental group were added to the constructs and incubated for three more days. Then the constructs were moved to a Polydimethylsiloxane (PDMS) device between a pair of carbon field electrodes, and analyzed in a 37°C environmental chamber at 4X magnification. Measurements were recorded using an inverted microscope (Olympus, Solent Scientific) mounted on a vibration isolation table. The electrical properties of the constructs were assessed by measuring the excitation threshold (ET), MCR (maximum capture rate), and amplitude of contraction (AC), as previously reported [23–25]. In brief, ET was the minimum electrical impulse (V/cm) required to induce synchronous contractions, and represents the electrical excitability of the constructs. For ET analysis, biphasic electrical stimuli were applied at a rate of 1 Hz starting at 2 V amplitude with 0.1 V increases until the entire construct started to beat synchronously; this point was designated as ET. MCR was the maximum beating frequency (Hz) attainable while sustaining the synchronous contraction, and is used as an indicator of the degree of cell integration and the capacity of the construct for synchronous beating. MCR was assessed by applying electrical stimuli at 2×ET amplitude, and increasing the stimulation frequency until the point that EHT lost the synchronous beating. AC reflects the changes in the surface area of the constructs between and during maximum contraction, and is used as a measure of the force of contraction. AC was measured as the relative changes in the surface area of the constructs following contraction. The area was measured on sequential images using ImageJ software (NIH), and the mean values of five consecutive contractions were used for analysis.

### Single cell preparation, flow cytometry analysis and cell sorting

Single cell suspensions were prepared by trypsinizing cells in plates or digesting patches with collagenase. For the latter, patches were incubated with 0.6 mg/mL collagenase type II (Worthington Biochemical, Lakewood, NJ) in culture medium for 15 min at 37°C and 15 min on ice with periodic pipetting. For intracellular staining, cells were fixed in 4% paraformaldehyde (PFA) for 15 min at RT, permeabilized with 0.25% Triton X-100 (15 min, RT), and stained with primary and secondary antibodies for 30 and 20 min, respectively. All antibodies were diluted in PBS containing 2% FBS and 0.1% Triton-X. Negative controls were incubated with isotype-matched control antibodies. Cells were analyzed on a Cytomics FC 500 (Beckman Coulter, Mississauga, ON) using FlowJo software (version 7.6.5, Tree Star, Ashland, OR). The following antibodies were used for MSC characterization: anti-CD73 (APC, 1:100, Biolegend), CD105, CD90, CD14 (PE, 1:100, BD Pharminogen), CD11b, CD19, CD34, CD45, and HLA-DR2 (PE, 1:100, Biolegend). The following primary and secondary antibodies were used in experiments for the expression of cardiomyocyte markers by rCM and MSC: mouse anti-myosin heavy chain (MyHC, 1:100, Abcam, Cambridge, MA), mouse anti-cardiac Troponin T (cTrpT, 1:100, Abcam), goat anti-mouse secondary antibody (APC-conjugated, 1:200, Biolegend). MSCs were detected by an anti-human CD44 antibody (FITC, 1:100, eBioscience, San Diego, CA). MSCs (CD44+) were isolated from the co-culture with rat cells using fluorescence-activated cell sorting (FACS) in a BD FACSAria machine with 20 psi, and 100 μm nozzle. The following antibodies were used for experiments with MSC/lymphocyte or MSC/monocyte co-cultures: anti-CD4 (FITC, 1:60, eBioscience), CD90 (PerCp-Cy5.5, 1:15, Biolegend, San Diego, CA), CD14 (PE-Cy7, 1:60, Biolegend), CD206 (PE, 1:30, Biolegend) and CD163 (FITC, 1:30, Biolegend). Expression of CD90 was used to gate out the MSCs (CD90+) in co-culture with monocytes.

### Proliferation and colony-formation assays

For MSC proliferation analysis, one day after culture or co-culture, cells were labeled with bromodeoxyuridine (BrdU, 50 μg/mL) for 48 h. Cells were harvested and stained with anti-CD44 antibody before BrdU staining. Cells were then fixed/permeabilized in 70% ice-cold ethanol (while vortexing) before being re-suspended in denaturing solution containing 2N hydrochloric acid (HCL)/1% Triton X-100 to produce single-stranded DNA. The residual acid was neutralized by 0.1 M sodium tetraborate (Na_2_B_4_O_7_, Sigma), pH 8.5. Next, cells were stained with rat anti-BrdU antibody (1:100, Abcam) for 20 min, washed and incubated with secondary antibody (PE-conjugated goat anti-rat, 1:2500, SouthernBiotech, Birmingham, AL) for another 20 min. Cells were washed and analyzed by flow cytometry, and double-positive (CD44/BrdU) MSCs were quantified. Proliferation of human dermal fibroblasts was studied by flow cytometry using the Click-iT® EdU Imaging Kit (ThermoFisher Scientific) following manufacturer’s instructions.

For CFU-F (colony-forming unit-fibroblast), FACS-sorted MSCs were cultured at three different densities in MesenCult MSC Basal Medium (Stem Cell Technologies) containing Mesenchymal Stem Cell Stimulatory Supplements (Stem Cell Technologies). A total number of 2.5×10^4^, 5×10^4^, and 1×10^5^ cells were plated on 150 mm culture dishes. After 14 days, colonies were visualized by Giemsa (Sigma) staining and those containing more than 40 cells were counted.

### Cell death assay

Cell death was induced by growing rCM/cFb either mono-cultured or co-cultured with MSC or human dermal fibroblast in serum-free media for 20 h before incubation with Paclitaxel/Taxol (Cedarlane, Burlington, ON) at a final concentration of 2.5 μM, 24 h for cells cultured on plate, and 10 μM, 30 h for cells cultured in collagen patches. Next, single cell suspensions were prepared and stained with anti-human CD44 antibody (APC, eBioscience) for negative selection of rat cells (CD44-ve) before staining with annexin-V using an ApoScreen Annexin-V kit (SouthernBiotech) following manufacturer’s instructions. Cell death was quantified in CD44(-ve) rat cells by flow cytometry.

### Immunostaining

Cardiac tissue constructs were fixed in 10% neutral buffered formalin (Sigma) at 4°C overnight before transferring to PBS. Fixed samples were sent to the Pathology Research Program at the University Health Network for paraffin embedding and sectioning (5 μm thickness). Slides were deparaffinized, processed for antigen retrieval by heat treatment for 20 min at 95°C in a decloaking chamber (Biocare Medical) followed by blocking with 3% bovine serum albumin (BSA, Bioshop, Burlington, ON) for 1 h, RT. The sections were incubated with the primary antibodies mouse anti-cTrpT (1:50, Abcam) and rabbit anti-human CD44 (1:50, Millipore) at 4°C overnight. Incubation with secondary antibodies FITC-conjugated goat anti-mouse (1:50, Sigma-Aldrich) and PE-conjugated goat anti-rabbit (1:40, Sigma-Aldrich) was performed for 1 h, RT. All antibodies were diluted in Tris Buffered Saline (TBS) containing 0.5% Tween 20 and 1% BSA, and all incubation steps were performed in a humidified chamber. The sections were counterstained and cover-slipped using 4,6-diamidino-2-phenylindole (DAPI)-containing mounting media (Invitrogen), and analysis was performed using a fluorescent microscope (Olympus. Solent Scientific).

### Gene and protein expression analyses, cytokine quantification

Total RNA was isolated using TRIzol (Invitrogen), and reverse transcribed using a High Capacity cDNA Transcription Kit (Applied Biosystems, Burlington, ON) following manufacturer’s instructions. Quantitative real-time PCR (qPCR) was performed using a SYBR® Green kit (Applied Biosystems) with human- or rat-specific primers on 7900HT Fast Real Time PCR System (Applied BioSystems). Samples were tested in duplicate and the mean values from independent experiments were used for analysis. A reaction with no cDNA template was used as negative control. Relative gene expression in rat or human cells was calculated by the 2-ΔCt method using glyceraldehyde 3-phosphate dehydrogenase (GAPDH, also species-specific) as internal control. Primer sequences are presented in S1 Table.

Proteins were isolated from cell lysate by scraping cells with non-reducing sample buffer. The proteins were equally loaded (based on BCA Protein assay kit, Invitrogen) and run on reducing 10% SDS-PAGE gel followed by wet transfer onto nitrocellulose membrane. The membrane was blocked with 5% non-fat skim milk for 1 h, followed by incubation with primary antibodies against alpha-smooth muscle actin (α-SMA) (mouse, a kind gift of Giulio Gabbiani, University of Geneva, Switzerland) and vimentin as internal control (mouse, Dako, Burlington, ON). After brief washing with (1XTBS, 0.1% Triton-X100), primary antibodies were detected by anti-mouse-680nm fluorescently labeled secondary antibodies, and detected using a LiCor Fx Imaging system (LiCor Biosciences, Lincoln, NE). Band intensities were semi-quantified using Image Studio (LI-COR Biosciences) and normalized to that of vimentin.

For cytokine analysis, MSCs were cultured in plates or collagen patches for 3 days before the medium was changed and cells were incubated for another 24h before the analysis. The amount of secreted cytokine/chemokines was measured in cell-free supernatants by sandwich ELISA using human-specific kits (R&D Systems, Minneapolis, MN) following manufacturer’s instructions. Samples were tested in duplicate and the mean value of each duplicate was used for analysis. The amount of cardiac trophic factors in the culture medium was determined using a membrane-based antibody array system (RayBiotech, Norcross, GA). Signals were semi-quantified by densitometric analysis and expressed relative to control medium.

### Cell contraction assay

Cellular contractility was assessed using deformable silicone substrates that wrinkle under cell force exertion as described previously [26]. Polydimethylsiloxane Sylgard 184 (Dow Corning) was polymerized into 35 mm petri dishes at 60°C to yield wrinkling substrates with Young’s modulus of 5 kPa. Substrate surfaces were activated by vacuum plasma oxygenation (PE-100, Plasma Etch, Inc., Carson City, NV) for 25 sec and then coated with (10 μg/mL) human plasma fibronectin (Millipore) overnight at 37°C. MSCs were grown in either collagen-coated plate or collagen patches for 3 days. Cells treated with transforming growth factor-beta (TGF-β1) or IFN-γ were used as positive and negative control, respectively. Then, cells were harvested and plated onto wrinkling substrates for 4 h at a density of 2.5×10^3^ cells/cm^2^. Live phase contrast images of substrate wrinkling were acquired using 10X and 20X objectives mounted on a Zeiss Primo Vert microscope (Carl Zeiss Microscopy). Wrinkling was quantified using Fiji (NIH image analysis software). Briefly, 8-bit monochrome images were auto-thresholded, masked and analyzed with the following parameters (size: 0-infinity and circularity: 0–0.3). Relative contraction was quantified and expressed as wrinkling area/cells [27].

### Statistical analysis

Statistical analysis was performed using Prism4 (GraphPad) software. Results are expressed as mean ± SEM. Statistical significance was assessed using Student’s *t*-test (between two groups), one-way ANOVA (for more than two groups), and two-way ANOVA (for more than two groups between 2D and 3D cultures) using the Tukey-Kramer post hoc test. Fold change data were log-normalized before analysis. The significance was denoted with asterisks corresponding to the *P* value (*: P < 0.05, **: P < 0.01, ***: P < 0.001).

## Results

### Collagen scaffold enhances the ability of MSCs to express cardiac markers in co-culture with neonatal rat cardiomyocytes

**Fig 1:**
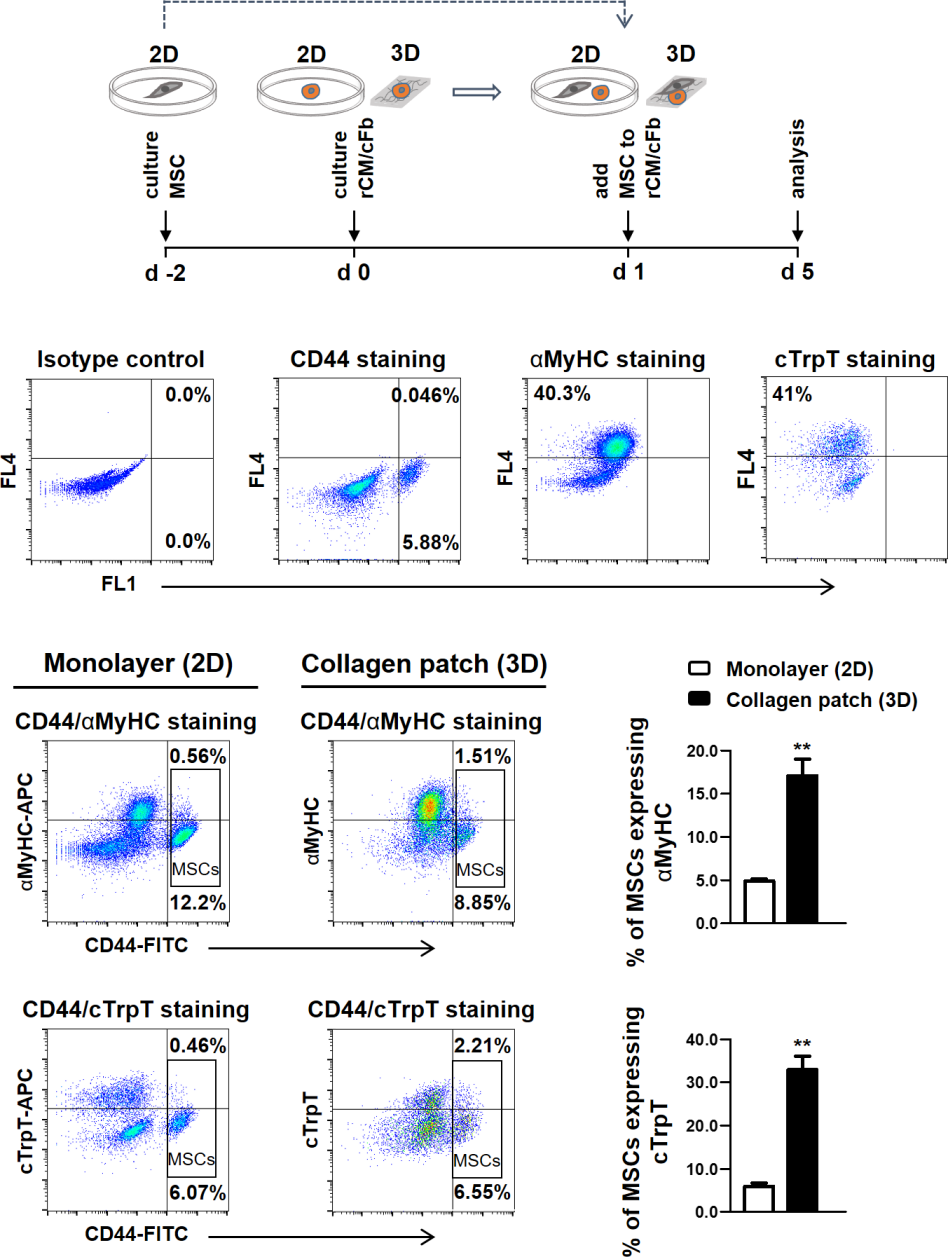
Expression of cardiac markers by human MSCs co-cultured with neonatal rat cardiomyocytes. Schematic timeline of the experiment is shown at the top. Flow cytometry analysis showed enhanced expression of α-myosin heavy chain (αMyHC, n = 4 MSC donors) and cardiac troponin-T (cTrpT, n = 3 MSC donors) in MSCs (CD44+) co-cultured with neonatal rat cardiomyocyte and cardiac fibroblasts in patches. Co-cultured cells were double-stained with FITC-conjugated anti-human CD44 and APC-conjugated anti- αMyHC or -cTrpT antibodies. Top row panels show isotype and single staining of the co-cultured cells. Error bars are SEM. * represents the statistical difference between groups (**P <0.01).

MSC phenotype was probed by the expression of mesenchymal markers and lack of the expression of hematopoietic markers along with the ability to differentiate to adipogenic, osteogenic and chondrogenic lineages (S2 Fig). The purity of cardiac cell extracts was assessed by the expression of cardiomyocyte markers alpha-myosin heavy chain (αMyHC) and cardiac troponin T (cTrpT) and the isolates containing a minimum of 70% rat cardiomyocytes (rCM) were used for the experiments (S3 Fig). We co-cultured MSCs with rCM/cardiac fibroblast (cFb) in plate or collagen patches and investigated the expression of cardiac-specific proteins by MSCs. At day 5 of co-culture, some of CD44(+) MSCs expressed cardiomyocyte sarcomeric proteins MyHC and cTrpT, which were absent in control MSCs, suggesting acquisition of a cardiomyocytic phenotype (S4A Fig). The number of MSCs expressing cardiac markers was significantly higher in collagen patch than in the culture plate (17.1 ± 1.9% in patch vs. 4.8 ± 0.2% in plate for MyHC, *P<* 0.01 and 32.9 ± 3.1% in patch vs. 5.9 ± 0.7% in plate for cTrp-T, *P<* 0.01) (Fig 1) indicating the enhanced ability of MSCs to acquire a cardiac cell-like phenotype in collagen scaffold. We also investigated a possible role for the increased progenitor activity of MSCs in collagen scaffold as a mechanism for the observed enhanced ability of these MSCs to undergo a partial cardiomyogenic reprogramming by examining the proliferation and colony forming ability of MSCs in our cultures. MSCs maintained in collagen patches were smaller than those cultured on plates (S4B Fig). BrdU incorporation assay showed significantly lower proliferation in MSCs in patches; proliferation rates increased after co-culture with rCM/cFb in both plates and patches but always remained lower in the latter. These results were not due to an increase in the frequency of the slow-growing progenitor cells in patches; CFU-F activity of MSCs was always similar between MSCs cultured in plates or patches (S4C and S4D Fig).

### MSCs do not affect the electrical excitation and contraction of cardiac tissue constructs

**Fig 2:**
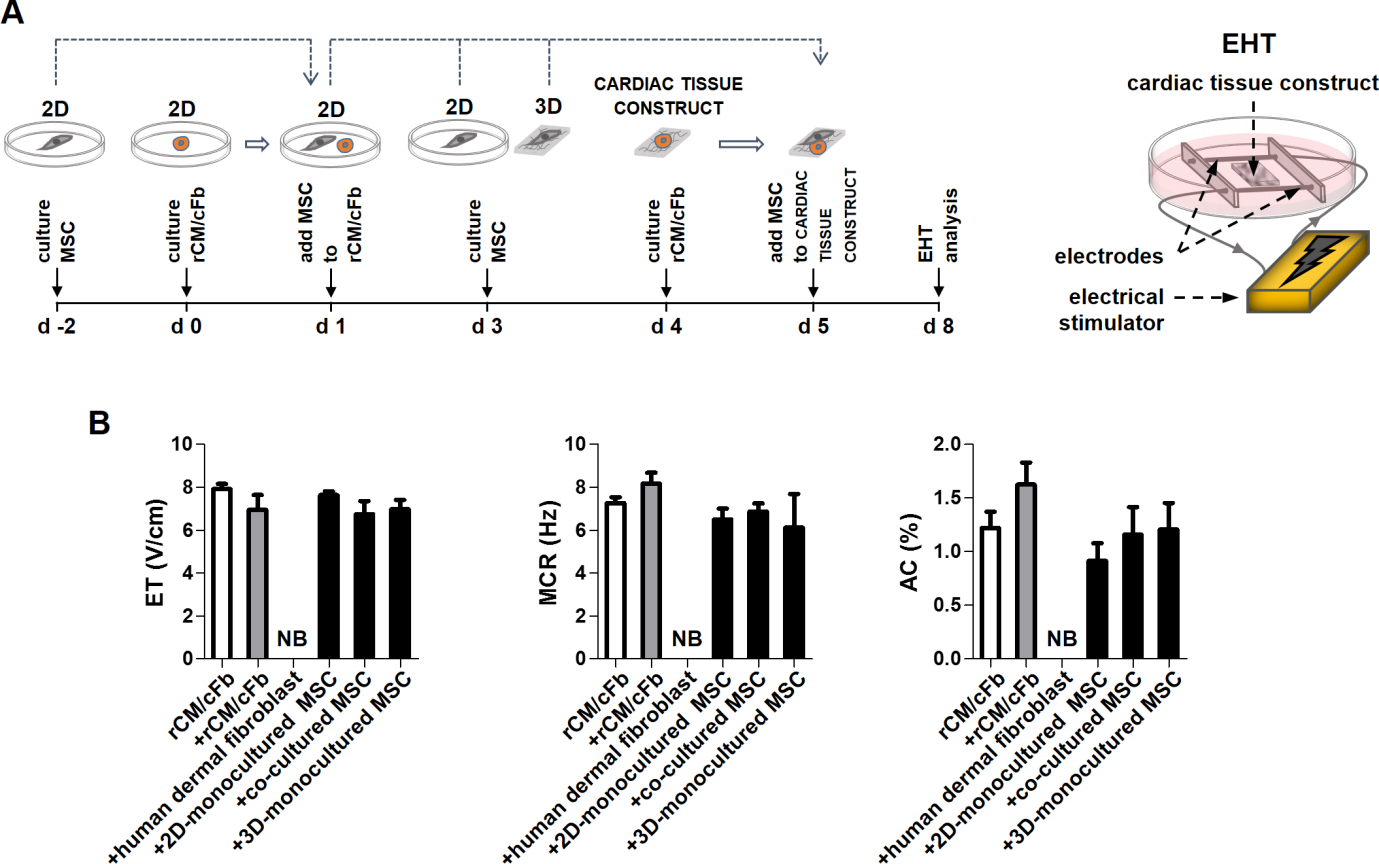
Electro-functional analysis of engineered heart tissue constructs. A) Schematic illustration of the experimental design and engineered heart tissue (EHT) model. B) ET (excitation threshold), MCR (maximum capture rate) and AC (amplitude of contraction) of the constructs before and after addition of MSCs were measured. Constructs with similar number of rat cardiomyocyte/cardiac fibroblast (rCM/cFb) were used as control (+rCM/cFb group). Addition of human dermal fibroblasts turned constructs into non-beating (NB) patches. Addition of MSCs either mono-cultured in plates (2D) or collagen patches (3D) or co-cultured with rCM/cFb in plates did not affect the functional parameters of the constructs (n = 4–8). Error bars represent SEM.

We investigated the effect of MSCs on cardiac tissue function by adding MSCs isolated from mono-cultures in plates or patches or from co-culture with rCM/cFb in plates to EHT (Fig 2A). Our attempts to isolate MSCs co-cultured with rCM/cFb in patches (which contained higher number of cardiac protein-expressing MSCs) did not yield sufficient number of viable cells for this analysis after enzymatic digestion of the patches and FACS sorting of the single cell preparations. As controls, we seeded EHT constructs with equal number of human dermal fibroblast (negative control) and rCM/cFb (positive control). Addition of rCM/cFb moderately improved the functional parameters of EHT constructs; this group, containing total cell numbers similar to other experimental groups, was used as baseline. MSCs, either from mono-cultures or pre-cocultured with rCM/cFb, had no adverse effect on the electrical excitability and contractility of EHT constructs. Unlike MSCs, addition of human dermal fibroblasts resulted in loss of synchronous beating of the constructs (Fig 2B). These results indicate that, unlike dermal fibroblasts which adversely affected the function of EHT constructs, MSCs do not interfere with electrical signal conduction and/or contraction in the cardiac tissue. The lack of functional changes after addition of MSCs however, even after prior co-culture with rCM/cFb, some of which expressed sarcomere proteins, indicates that MSCs do not directly contribute to the excitability and contraction of myocardium.

### MSCs preserve anti-apoptotic effect and enhance expression of cardiotrophic factors in collagen scaffold

**Fig 3:**
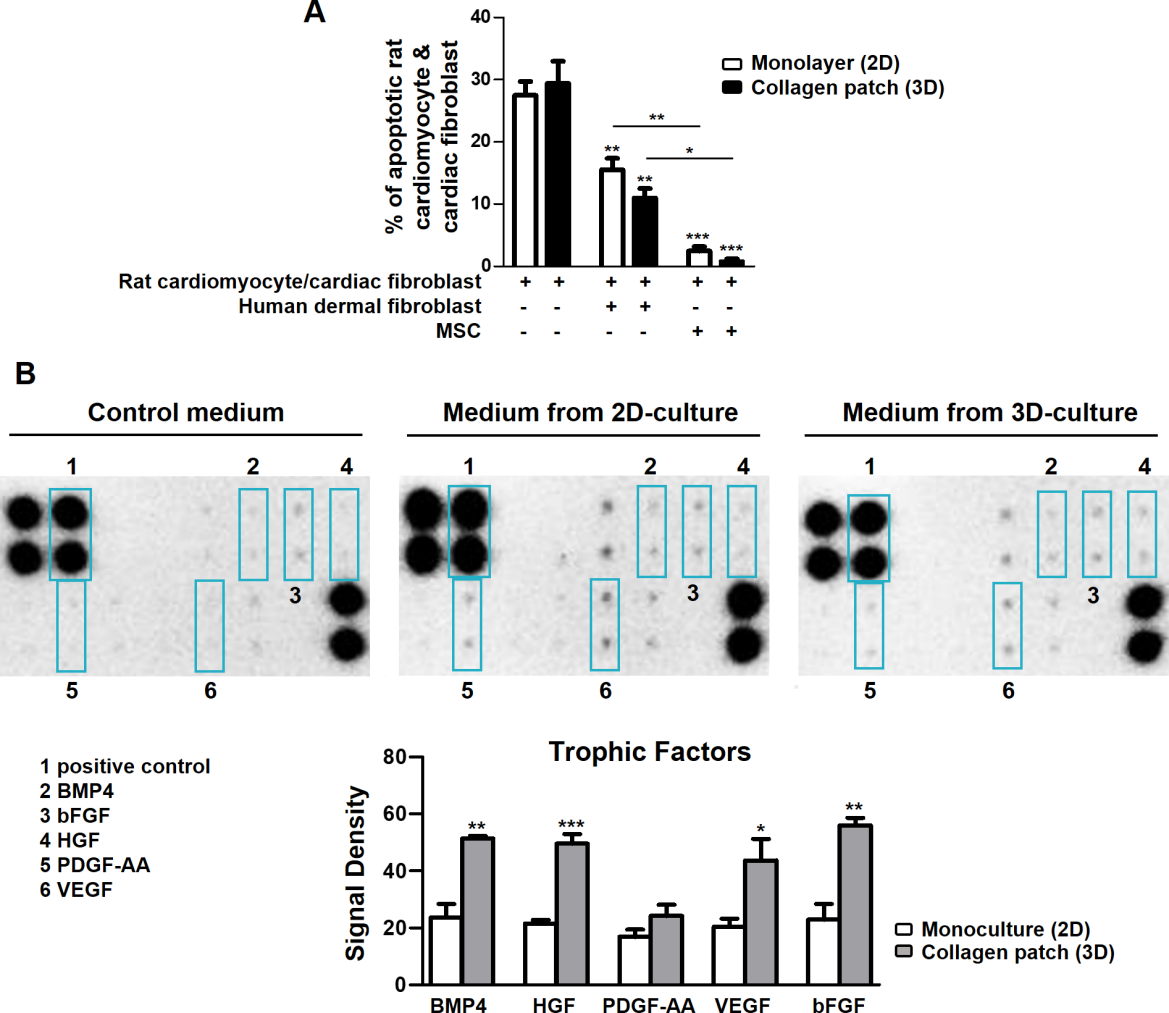
The anti-apoptotic effect and secretion of trophic factors by MSCs in plate (2D) and collagen scaffold (3D). A) The proportion of apoptotic rCM/cFbs was decreased after co-culture with human dermal fibroblasts (n = 3 replicates) and more significantly with MSCs (n = 3 MSC donors). B) MSCs were cultured in plates or patches for 3 days before the medium was changed and cells were incubated for another 24h before the analysis. The amounts of proteins in culture medium quantified by antibody array normalized to the medium containing no cells showed enhanced secretion of BMP4 (bone morphogenic protein 4), HGF (hepatocyte growth factor), VEGF (vascular endothelial GF) and bFGF (basic-fibroblast GF) by MSCs grown in collagen patches (3D) compared to those cultured in plates (2D) (n = 3 MSC donors). PDGF, platelet-derived GF. Error bars are SEM. * represents the statistical difference between groups (*P <0.05; **P <0.01; ***P <0.001).

MSCs promote tissue repair via several mechanisms including inhibition of cardiomyocyte apoptosis and secretion of cardiotrophic factors [28,29]. To study the anti-apoptotic effect of MSCs in 2D and 3D cultures, cell death was induced in rCM/cFb in the presence or absence of MSCs, and the frequency of apoptotic cells were analyzed by flow cytometry in non-MSC (CD44-ve) rCM/cFb cells. Co-cultures of rCM/cFb with human dermal fibroblasts served as controls. Co-culture with either human dermal fibroblasts or MSCs decreased apoptosis of rCM/cFb, but the difference was more significant in MSC co-cultures (Fig 3A). However, there was no difference in the frequencies of apoptotic rCM/cFbs co-cultured with MSCs, either in plates or patches, indicating that the pro-survival effect of MSCs is preserved in the latter. These data indicate a prominent pro-survival effect of MSCs that is preserved in 3D collagen patches.

The expression of trophic factors by MSCs was assessed by both qPCR and an antibody array system. MSCs in collagen patches showed increased transcription of bone morphogenic protein 4 (BMP4), hepatocyte growth factor (HGF) and vascular endothelial growth factor (VEGF) whereas the expression of PDGF-AA remained unchanged between cultures (S5 Fig). These results were confirmed by an antibody array which showed higher levels of BMP4, HGF, VEGF, and basic fibroblast growth factor (b-FGF) secreted into culture medium by MSCs cultured in patches (Fig 3B). These data suggest that collagen scaffold may enhance the cardiac repair potential of MSCs by inducing the secretion of cardiotrophic factors.

### MSCs in collagen scaffold exhibit less myofibrogenic phenotype

**Fig 4:**
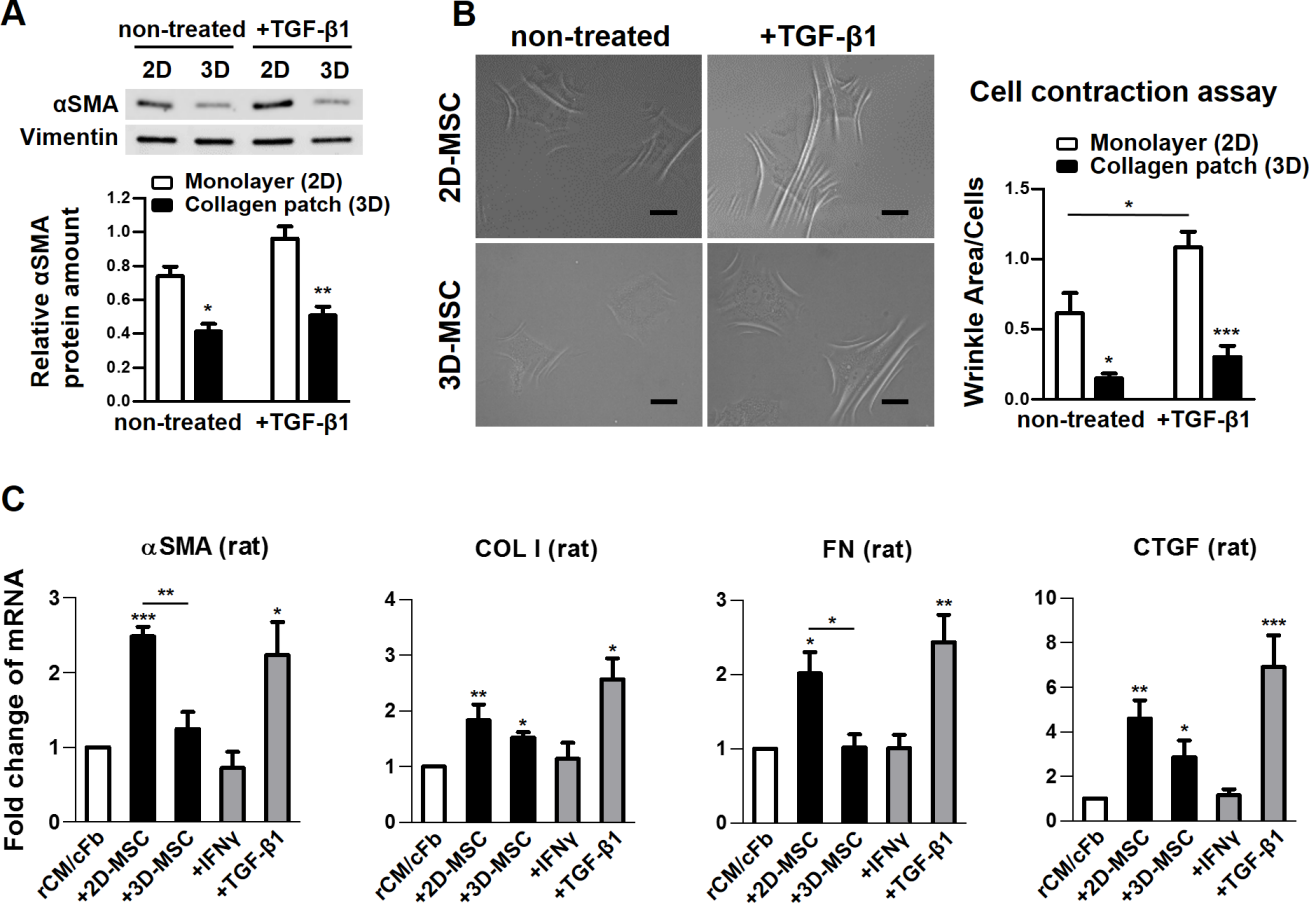
Anti-fibrotic properties of MSCs cultured in plate (2D) or collagen scaffold (3D). Cells were maintained in plates or collagen patches for 3 days to establish the respective phenotype for each analysis. A) Cellular αSMA content analyzed by Western blot (n = 3 MSC donors) and B) cell contractility on silicon substrate (n = 4–5 replicates of 3 MSC donors) was reduced in MSCs grown in patches. *Scale bar 20 μm*. C) The increase in gene expression by neonatal rat cardiomyocytes and cardiac fibroblasts (rCM/cFb) after co-culture with MSCs was either absent or less significant in collagen patches (n = 4 MSC donors). rCM/cFbs treated with TGF-β1 and IFNγ were used as positive and negative control, respectively. αSMA, alpha-smooth muscle actin; COLI, collagen type I; FN, fibronectin; CTGF/CCN2, connective tissue growth factor. Error bars are SEM. When not specified by a line, * represent the statistical difference within groups or in comparison to the control/white bar (*P <0.05; ** *P* <0.01; ***P <0.001).

Culture of MSCs on conventional 2D substrates progressively induce a fibrogenic phenotype [30,31] that potentially impairs the therapeutic value of MSCs [32]. Hallmarks of MSC fibrogenesis are neo-expression of αSMA, leading to higher isometric contraction and enhanced ECM production. We next studied the expression of ECM components and the development of this myofibrogenic phenotype by MSCs cultured in monolayer or in collagen patches. MSCs treated with TGF-β1 were used as control for maximum activation of the pro-fibrotic phenotype. Gene expression analysis showed that the baseline expression of pro-fibrotic genes, i.e. αSMA, CCN2 (connective tissue growth factor/CTGF), and FN (fibronectin) was significantly reduced in MSCs cultured in collagen patches compared to those cultured in collagen-coated plates. Gene expression was upregulated following treatment with TGF-β1, however the mRNA levels remained significantly lower in patches (S6 Fig). The reduced αSMA content in MSCs cultured in collagen patches was also confirmed at the protein level (Fig 4A). Functionally, MSCs cultured in patches exhibited reduced isometric contraction on deformable silicone substrate that wrinkle upon high cell force exertion (Fig 4B). Next, we looked at the expression of the fibrosis-associated genes by rat cells before and after co-culture with MSCs. In rCM/cFb, we found an increase in the expression of αSMA, COLI (collagen type-I), FN and CCN2/CTGF transcripts after co-culture with MSCs, which was absent or less significant in patches (Fig 4C). Taken together, these data indicate that MSC culture in collagen patches suppresses development of myofibrogenic features, which would be beneficial for cardiac repair by decreasing deposition of ECM and contracture into scar tissue either from MSCs or MSC-educated cFbs.

### MSCs exhibited comparable paracrine activity but an attenuated response to LPS stimulation in collagen scaffold

**Fig 5:**
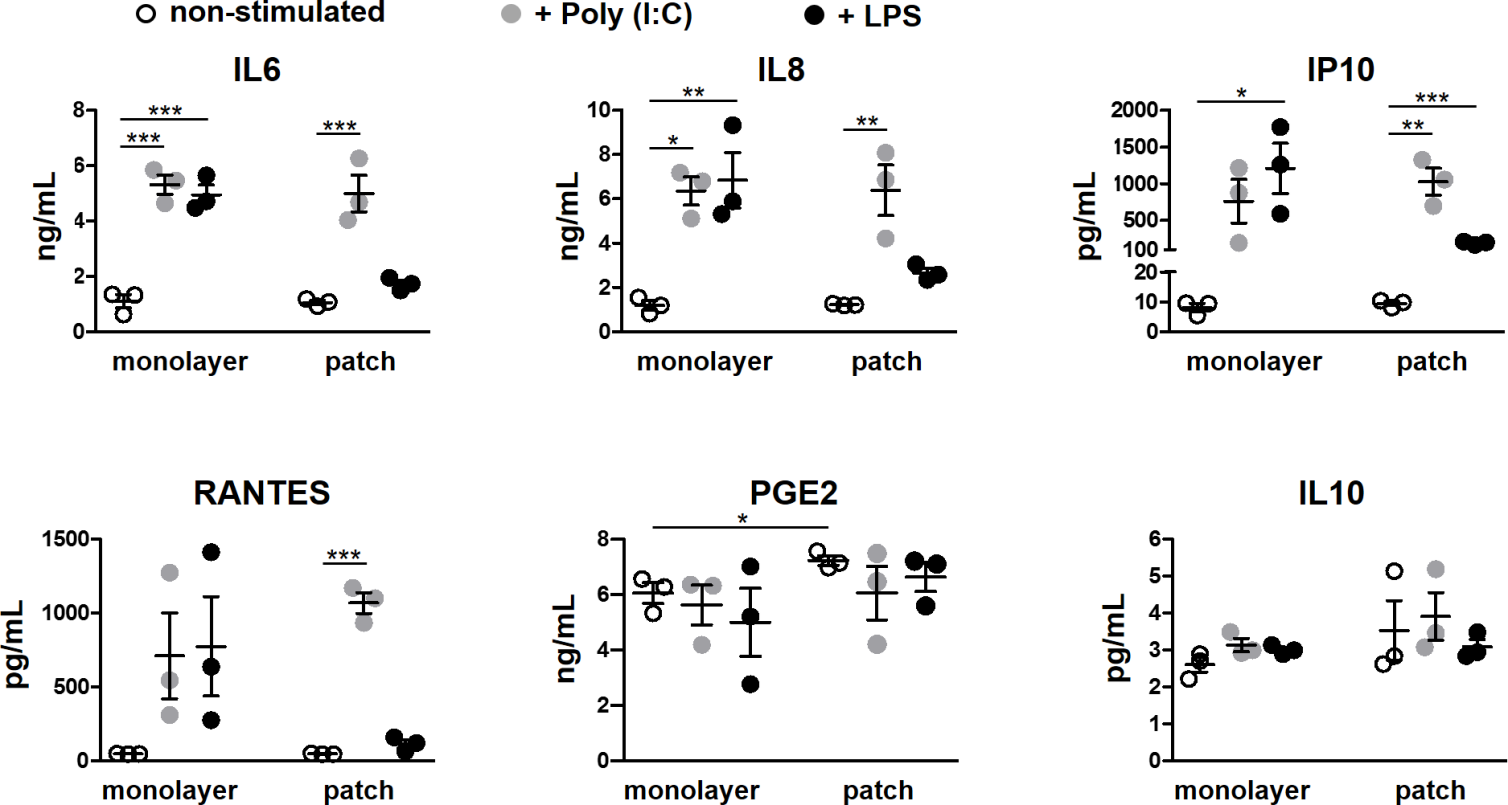
Secretion of soluble factors by MSCs grown in plate or collagen scaffold. MSCs were maintained in plates or collagen patches for 3 days before respective treatment and subsequent analysis. The basal secretion of cytokines/chemokines into culture medium (measured by ELISA) was similar by non-stimulated MSCs maintained in plates or in patches. The basal level of prostaglandin (PG) E2 was slightly, but significantly, higher in patch-MSC cultures. In plates, stimulation of MSCs with Poly (I:C) or LPS increased the level of IL6, IL8, IP10 and RANTES. In path-cultured MSCs, Poly (I:C) induced the secretion of IL6, IL8, IP10 or RANTES but the response to LPS was either absent or decreased. Stimulating MSCs with either Poly (I:C) or LPS had no effect on PGE2 or IL10 production by MSCs. In all experiments n = 3 MSC donors. Error bars are SEM. * represents the statistical difference between groups (*P <0.05; **P <0.01; ***P <0.001).

Several studies have reported increased anti-inflammatory properties of MSCs in 3D cultures. The paracrine activity of MSCs is important for their regenerative function mainly via their interaction with various cells from the immune system which are present at the site of tissue injury and play a role in tissue repair. We examined the secretion of immune mediators by MSCs in our cultures and looked at their ability to mount a response when stimulated with inflammatory signals mimicked by Poly (I:C) and LPS, TLR 3 and 4 agonists, respectively. MSCs expressed TLR3 and TLR4 at comparably high levels in plate and patch, and activated the NFκB pathway after stimulation with Poly (I:C) or LPS (S7A and S7B Fig). MSCs maintained in plate or collagen patch had similar constitutive expression of genes with pro-inflammatory effects such as IL(interleukin)-6, IL-8, RANTES and IP-10, or anti-inflammatory function including LIF (leukemia inhibitory factor), COX-2 (PTGS2/prostaglandin-endoperoxide synthase 2), TSG-6 (TNFAIP6/tumor necrosis factor-inducible gene 6 protein), IDO (indoleamine 2,3-dioxygenase 1) and IL-10 (S7C Fig). In line with these data, the amounts of IL-6, IL-8, RANTES and IP-10 secreted into the medium by MSCs was also comparable between monolayer and patch cultures. Of factors with anti-inflammatory effects, the basal level of IL-10 was similar in monolayer and patch cultures while the amount of PGE2 was slightly, but significantly, higher in culture medium of MSCs in collagen patches (Fig 5).

Incubation with either Poly (I:C) or LPS significantly increased the transcription of pro- and anti-inflammatory genes by MSCs in the same manner in both cultures (S7D Fig). MSCs also comparably increased the secretion of IL6, IL8, RANTES and IP10 into medium after stimulation with Poly (I:C) in plate or patch cultures. LPS stimulation, however, produced different results. In monolayer culture in plate, LPS stimulation triggered a trend or significant increase in the secretion of these factors by MSCs whereas in patch culture the level of cytokines released into the medium by MSCs after LPS challenge increased to a lesser degree or remained unchanged (Fig 5). Of factors with known anti-inflammatory effects, GM-CSF was not detectable in the culture medium of MSCs even after TLR 3 or 4 activation and the IL-10 level was very low and did not change with Poly (I:C) or LPS stimulation. Similarly, we did not find any changes in PGE2 secretion after stimulation with these ligands in our cultures. Taken together, these data indicate that the ability of MSCs to secrete factors with paracrine activity are preserved in collagen scaffold while the attenuated response to LPS stimulation suggest a decreased pro-inflammatory response by MSCs in collagen scaffolds upon encounter with certain stimulatory signals.

### Immune-suppression and macrophage polarization by MSCs in 2D and 3D cultures

**Fig 6:**
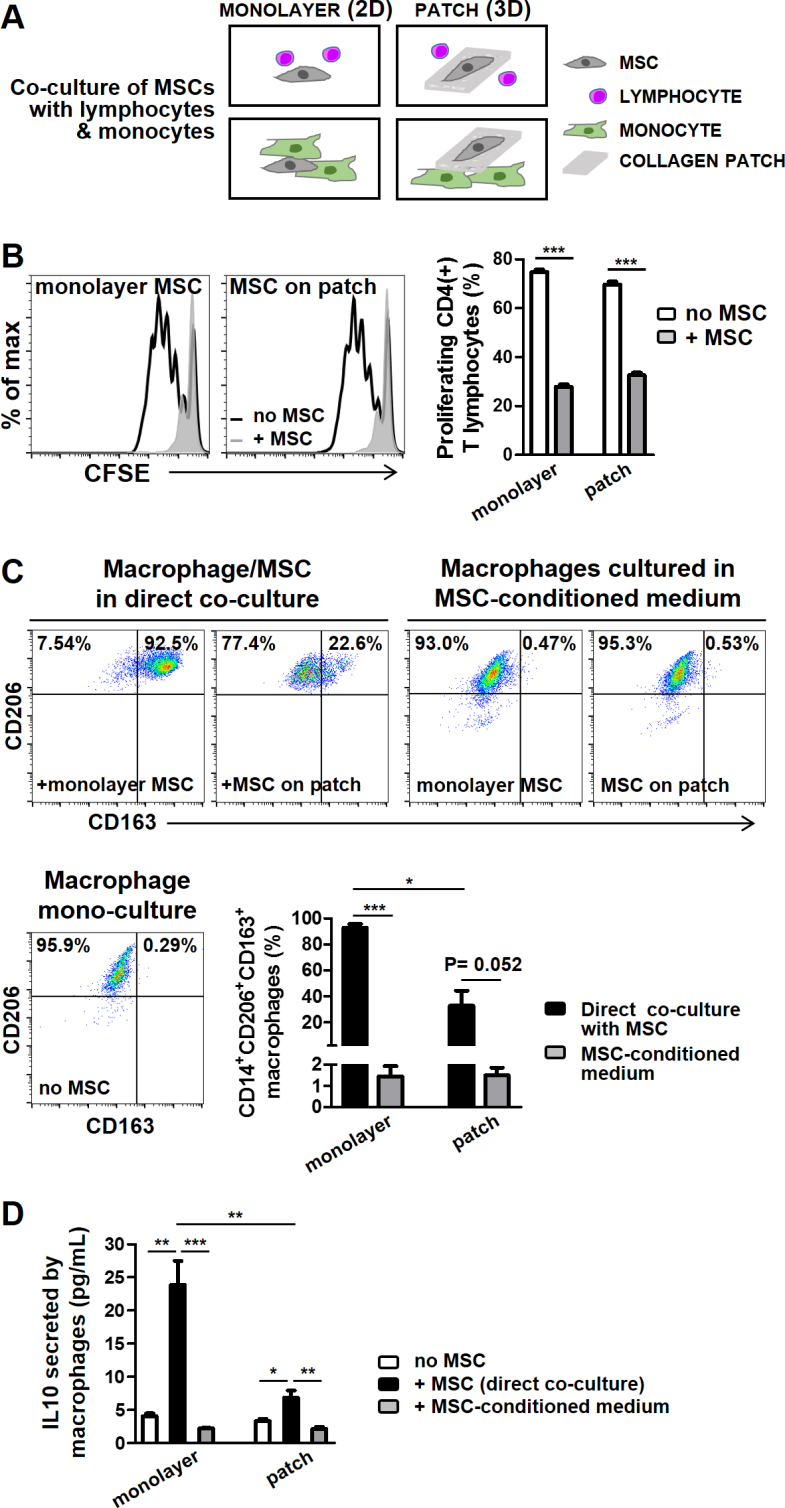
Immune function of MSCs in plate and collagen scaffold. A) Schematic illustration of co-culture experiments. B) MSCs maintained in plates or collagen patches similarly suppressed the proliferation of CD4(+) lymphocytes. C) Induction of M2 macrophages by MSCs was reduced when MSCs were cultured in collagen patches and was absent in MSC-conditioned medium. D) The increase in IL10 secretion in macrophage/MSC co-cultures was less significant with MSCs cultured in patches, and was absent in monocytes cultured in MSC-conditioned medium. In all experiments n = 3 MSC donors. Error bars are SEM. * represents the statistical difference between groups. (*P <0.05; ** *P* <0.01; ***P <0.001).

We next studied the modulatory function of MSCs, the inhibition of lymphocyte proliferation as a classic indicator of the immunosuppressive effects of MSCs as well as their effect on macrophage polarization, in co-culture with allogeneic lymphocytes and monocytes in 2D and 3D cultures (Fig 6A). In co-culture with CD4(+) lymphocytes, MSCs significantly inhibited T cell proliferation regardless of whether they were cultured as monolayer or in collagen patch (Fig 6B). In co-culture with CD14(+) monocytes, MSCs activated reprogramming towards an anti-inflammatory, pro-regenerative CD206(+)CD163(+) M2 phenotype in both cultures, however, the frequency of M2 macrophages was lower when MSCs were cultured in collagen patches. There was no difference in the viability of CD4(+) T lymphocytes or CD14(+) macrophages in co-culture with MSCs in 2D and 3D cultures (S8 Fig). The lower frequency of M2 macrophages in our 3D culture could be a result of limited cell-cell contact between MSCs and monocytes that were, respectively, cultured in patches and the bottom of the wells. Alternatively, it could be due to an attenuated inherent ability of MSCs in collagen patches to induce such differentiation. The role of cell contact was investigated in the experiments with MSC-conditioned medium derived either from monolayer- or patch-cultured MSCs, which failed to activate CD206(+)CD163(+) M2 macrophages, supporting a role for direct cell-cell contact in macrophage polarization by MSCs (Fig 6C). Consistent with these data, the IL-10 level was significantly lower in the supernatant of monocytes/macrophages cultured in MSC-conditioned medium or co-cultured with MSCs that were cultured in collagen patches compared with monolayer co-cultures in which both monocytes and MSCs were in direct cellular contact on plates (Fig 6D). We did not distinguish the cellular source of IL-10 in our co-cultures but given that we did not detect high levels of IL-10 in MSC mono-cultures, we considered M2 macrophages being the most likely source of IL-10 in these co-cultures. These data suggest that the immunosuppressive function of MSCs is preserved in collagen patch and that direct cell contact or, at least, crosstalk between MSCs and monocytes is important for activation of M2 macrophages. Altogether, these results indicate that MSCs maintain the paracrine activity and immunomodulatory function in 3D collagen patches and develop less pro-inflammatory phenotype when activated by TLR4 agonist compared with their monolayer counterparts cultured in plates.

## Discussion

Cardiac repair has been investigated with a variety of cell types, including MSCs [33]. Clinical trials of MSCs for cardiac disease, however, lack the expected efficacy [34–36], which warrants a search for conditions that might enhance their regenerative potential. Consequently, a better understanding of the mechanisms by which MSCs mediate tissue repair is necessary and could be facilitated by studying conditions similar to those *in vivo*. Here, we investigated MSCs maintained in collagen scaffold (as a 3D model), a cell product already used in clinical trials for cardiac repair [3]. We were primarily interested in the ability of MSCs to acquire a cardiac cell-like phenotype and to investigate the electromechanical contribution of these partially reprogrammed MSCs that expressed several cardiac proteins. We showed that in collagen patches, MSCs could enhance the expression of cardiomyocyte-specific proteins but did not change the function of the cardiac tissue constructs. MSCs, however, did not interfere with electrical signal propagation and contraction of cardiac tissue constructs, in contrast to dermal fibroblasts which abolished the function when added to the constructs. MSCs in collagen patches secreted higher levels of cardiotrophic factors and developed a less myofibrogenic phenotype. Stimulation with either TLR3 or TLR4 agonists increased the expression of both pro- and anti-inflammatory factors by MSCs but pro-inflammatory responses were attenuated in patches. Altogether, these data suggest that MSCs develop a more pro-regenerative phenotype in collage scaffold.

The use of bioengineered heart tissue enabled us to perform functional analysis *in vitro* under conditions more reflective of the mechanical properties of myocardium *in vivo*, as reported by Bhana et al. [37] who showed that cardiac tissue constructs within biological range of stiffness of cardiac muscle tissue demonstrate morphological and functional phenotype similar to native myocardium. We also showed previously that the modulus of the collagen patches in our system is in the physiological range for neonatal rat ventricle stiffness and was not affected by cell cultivation [38]. Moreover, the absence of immune cells in our model allowed for studying the properties of MSCs important for cardiac repair independent of their well-established immunological interactions.

The differential phenotypes of MSCs in our cultures are mainly attributable to the biophysical properties of the 2D and 3D cultures. ECM stiffness, as an essential property of the microenvironment cells interact with, is a key factor influencing cell behavior and determining cell fate. The molecular mechanisms by which changes in matrix stiffness regulate cell fate including lineage commitment are largely unknown. The increased capacity of MSCs for cardiomyogenic reprogramming in 3D collagen patches in our studies is in agreement with previous reports using other 3D culture models [39,40]. Guan et al. [41] showed that 3D constructs that mimic the structure and biomechanics of heart tissue increase the efficiency of cardiomyogenic reprogramming of MSCs. The differential expression and/or molecular configuration of ECM proteins in 3D substrates may influence the cardiac lineage specification via integrin receptors and activation of the Wnt pathway [42,43]. In one study, MSCs formed spheroids and enhanced the expression of cardiac-specific genes on chitosan membranes compared to tissue culture plates, possibly through upregulating Wnt11 expression [40]. We also found that the expression of Dickkopf Wnt pathway inhibitor 1 (DKK1) was significantly decreased in patch-cultured MSCs (data not shown). Nevertheless, enhanced capacity of MSCs to acquire a cardiomyocyte-like phenotype in collagen patch is not the result of a selective increase in the number and/or activity of progenitor cells, as the CFU-F frequency of MSCs was similar whether the cells were cultured in plates or collagen patches.

We showed that MSCs had no effect on the functional properties of cardiac tissue constructs. This finding is in line with our previous study [14]. We showed no excitability in mouse MSCs that expressed cardiac markers in co-culture with embryonic rat cardiomyocyte, indicating that the partial reprograming of MSCs did not extend to the acquisition of electrical properties. These studies, however, are in contrast to the study by Serrao et al. [44] who modeled non-functional myocardium by developing constructs with half the number of cardiomyocytes compared with controls, and showed that addition of rat MSCs improved the function of these myocyte-depleted constructs. Nevertheless, the experimental design of their study differed considerably making it difficult to compare the results. Further, in the study by Serrao et al., the indirect comparison between the baseline control and the constructs with rat dermal fibroblasts (with no known functional benefit to myocardium) suggests functional improvement in the latter group whereas in our study, dermal fibroblasts had an adverse effect when added to the constructs. The latter finding indicates an important function of MSCs. Unlike fibroblasts, MSCs do not interfere with electrical conduction and signal propagation across the cardiac tissue constructs. Given the risk of arrhythmias in the cell therapy of cardiac diseases, this is particularly important for the clinical application of MSCs, especially when large numbers of cells (e.g. seeded in patches) engraft to host myocardium.

It is noteworthy that in our study only about five percent of the MSCs primed in co-culture with rCM/cFb expressed cardiac markers, which may be insufficient to induce significant changes in the electromechanics of the constructs to a level detectable by our model. Also, the fact that our model represented intact cardiac tissue with already optimal functionality may have obscured any further functional improvements. Nevertheless, our data infer that the reported therapeutic benefits of cardiomyogenic priming of MSCs [17–19,21] is more likely through mechanisms other than a direct contractile contribution by MSCs. One such mechanism may involve gap junction formation important for establishing intercellular coupling with host myocardium [45,46]. In MSCs pre-committed to the cardiac lineage, expression of gap junction proteins is increased [45], which may have an augmenting effect on the structural integration of MSCs with cardiac muscle. Moreover, cardiac pre-specification may affect the paracrine activities of MSCs to favor a more cardiotrophic phenotype by increasing the secretion of pro-survival and angiogenic factors and activating the intracellular signaling pathways that may enhance their long-term engraftment [17,47].

The interaction between MSCs and cells of the immune system is important at various stages of tissue remodeling and repair. The finding that MSCs maintained their paracrine activity and immunosuppressive function in collagen patch is important for bioengineering and the topical administration of MSCs in patches. The reduced secretion of inflammatory cytokines/chemokines following LPS stimulation by patch-cultured MSCs may be due to differences in the regulatory mechanisms including differential expression of inhibitors and/or adaptor molecules involved in TLR signaling in these cultures, and suggests that these MSCs may mitigate tissue damage via decreasing the recruitment of immune cells to the injured area. Further, the activation of M2 macrophages with anti-inflammatory/pro-regenerative functions in this study is important for MSC-mediated tissue repair and is consistent with our previous study in a mouse model of myocardial infarction [48]. However, the decrease in the frequency of alternatively activated macrophage in our 3D culture is likely a result of reduced MSC/monocyte cell contact, important for murine and human MSC-mediated macrophage polarization [49,50], and requires more investigation. In addition to the reduced pro-inflammatory response by MSCs cultured in collagen patches, the development of a less myofibrogenic phenotype by MSCs in patches independent of interactions with immune cells may also prevent excessive deposition of scar tissue-forming proteins after myocardial injury and promote a favorable remodeling. These findings, along with the enhanced production of cardiotrophic factors by patch-cultured MSCs suggest that delivering MSCs in collagen patches provides not only structural support to damaged myocardium but also promotes tissue repair and enhances regenerative potential of MSCs.

The cytokine response by MSCs after stimulation with TLR 3 and 4 agonists in this study are particularly interesting. We showed that stimulation of MSCs with either Poly (I:C) or LPS can lead to enhanced expression of pro-inflammatory cytokines. Also, activation not only of TLR3 but also of TLR4 on MSCs can promote an *anti-inflammatory phenotype* by upregulating the expression of anti-inflammatory factors. Previous studies have reported differential effects of TLR 3 and 4 activation on MSCs with a significant increase in the expression of anti-inflammatory immune mediators such as IDO or PGE2 by MSCs after TLR3 activation while the induction of these factors was either less significant or absent after stimulation with a TLR4 agonist, suggesting a pro- and anti-inflammatory phenotype of MSCs after respective activation of TLR4 and TLR3 [51,52]. Our findings, however, indicate a pleiotropic and probably context-dependent role of TLR 3 and 4 activation in the immune biology of MSCs. In line with these findings, we also recently showed that the activation of either TLR3 or TLR4 augments the induction of regulatory T cells by MSCs [53]. Taken together, these studies indicate that the currently held notion of TLR3 activation on MSCs leading only to anti-inflammatory responses and TLR4 ligation only to a pro-inflammatory profile, needs reassessment.

This study was not without limitations. The study would be improved by coating the surface of culture plates with matrigel and/or collagen to exclude their possible effect(s) on the observed phenotype in patches. However, although the effect(s) of biochemical and biophysical properties of the 3D culture cannot be extricated in our studies, and while the cell phenotype is most likely affected by both, we believe that the latter has a more significant impact on the observed phenotype in these studies. This is supported by a study in which MSCs, either cultured in plates pre-coated with collagen I or laminin or non-coated plates, showed similar expression of several lineage-specific genes (ACTC1/alpha cardiac muscle actin: cardiogenic, SPP/ostopontin: osteogenic, and PPARG/glitazone receptor: adipogenic) [54]. In the same study, ACTC1 was significantly upregulated in 3D collagen composites compared to 2D counterparts pre-coated with collagen I. Similarly, in another study, the structure of collagen substrates (i.e. hydrogel vs. sponge vs. membrane) had differential effects on the immunosuppressive and paracrine activities of MSCs [55]. Nevertheless, the regulatory effect of ECM, as a dynamic and interactive environment, on specific cell phenotype should be addressed in more sophisticated studies. EHT functional analysis might have been improved by testing the partially-differentiated MSCs co-cultured with rat cells in patches. Unfortunately, cell numbers obtained by FACS-sorting were insufficient; an alternative isolation method such as magnetic sorting may improve the cell yield. Alternatively, patches containing MSC co-cultured with rCM/cFb can be directly tested for electrical excitability and contractility and compared with monolayer cultures with extra measures to be taken to control for cell numbers because of the variations in proliferation rates in mono- and co-cultures. Another possible improvement involves the establishment of an EHT model that represents myocardial damage and cell loss. For instance, since *in vivo* the dead cardiomyocytes are replaced by cardiac fibroblasts, constructs with different proportion of these cells can be used to mimic varying degrees of myocardial damage. It is also noteworthy that, studies involving different species, although advantageous in many regards, are limited by the species-specific characteristics such as signaling pathways or bioactive molecules. This limitation, however, can, at least partially, be overcome by using lineage-specific human cells which have been made available by iPSC (induced pluripotent stem cell) technology. Finally, further studies of the regenerative properties of partially-differentiated MSCs may provide more insight into mechanisms underlying the enhanced therapeutic potency of these cells.

## Implications for cell therapy

The importance of partial differentiation of MSCs in tissue repair remain controversial. Our studies suggest that the functional recovery of the heart after MSC therapy is most likely not the result of a direct contractile contribution by MSCs. However, partial reprogramming may activate other cellular pathways that promote the repair process by MSCs. From a translational perspective, MSCs with a partial cardiac cell-like phenotype may undergo further reprogramming more readily when exposed to cardiogenic cues a second time after implantation, which may further improve their reparative function. Nevertheless, the lack of interference with the electrophysiology of myocardium remains an important consideration for cell therapy. Our studies suggest that adoption of a cardiac-cell like phenotype by MSCs does not cause aberrant electrical activity.

Optimum treatment for post-ischemic myocardial dysfunction involves approaches that support both the dynamics and the electrophysiology of damaged myocardium. With cell-based therapies, the inherent properties of cells and the delivery methods are important for efficient integration and functional recovery. These studies and similar reports showing the augmenting effects of 3D-based cultures suggest that the priming of MSCs by growing them on 3D platforms prior to clinical application may improve the efficacy of MSCs. In addition, bioscaffolds provide structural support and enhance cellular retention. Our studies suggest that a collagen scaffold may be a suitable vehicle for the MSC-based therapy of damaged myocardium by virtue of their enhancing effect on the regenerative properties of MSCs.

## Supporting information

S1 Table Primers used for real-time PCR. ACTA/αSMA, alpha smooth muscle actin; BMP4, bone morphogenic protein 4; CCL5/ RANTES, C-C motif chemokine ligand 5; COL1A1, collagen type 1 alpha 1; CTGF, connective tissue growth factor; CXCL10/IP10, C-X-C motif chemokine ligand 10; GAPDH, glyceraldehyde 3-phosphate dehydrogenase; HGF, hepatocyte growth factor; IDO1, indoleamine 2,3-dioxygenase 1; IL, interleukin; LIF, leukemia inhibitory factor; NFKBIA, nuclear factor kappa B inhibitor alpha; PDGFA, platelet derived growth factor subunit A; PTGS2/COX2, prostaglandin-endoperoxide synthase 2; TNFAIP6/TSG6, tumor necrosis factor-inducible gene 6 protein; VEGF, vascular endothelial growth factor; * rat-specific sequences.
(DOCX)

S1 Fig MSC mono- and co-cultures. MSCs were either mono- or co-cultured with other cells in plates or collagen patches. Rat cardiac cell extracts consist of cardiomyocyte and cardiac fibroblast (only cardiomycote is depicted in the figure). If applicable. MSCs underwent treatment before being washed and used in subsequent experiments. Monocytes were activated with GM-CSF (granulocyte macrophage colony stimulating factor) and lymphocytes were activated with CD3/CD28 beads and stained with CFSE (carboxyfluorescein succinimidyl ester) before co-culture with MSCs. Single cell suspensions were prepared by trypsinizing the cells in plates or digesting patches with collagenase.
(TIF)

S2 Fig Characterization of human bone marrow-derived MSCs. A) Flow cytometry analysis of MSCs showing the expression of CD73, CD105, CD90 and lack of the expression of hematopoietic markers CD11b, CD14, CD19, CD34, CD45, and HLA-DR2 by MSCs. Dashed lines are isotype controls. B) Tri-lineage differentiation of MSCs showing adipogenic (Oil Red O staining), osteogenic (Alizarin Red staining) and chondrogenic (Alician Blue staining). *Scale bar 50 μm*.
(TIF)

S3 Fig Purity of neonatal rat cardiac cell isolates. Rat cardiac cell extracts mostly contain cardiomyocytes expressing α-myosin heavy chain (αMyHC) and cardiac troponin T (cTrpT) proteins. Cardiac fibroblasts stain negative for cardiac markers. n = 3 independent isolations. Error bars are SEM.
(TIF)

S4 Fig Expression of cardiomyocyte-specific marker, proliferation and colony formation by MSCs. A) Immunohistochemistry staining showing the expression of cTrpT by MSCs co-cultured with neonatal rat cardiomyocyte/cardiac fibroblasts (rCM/cFb) in collagen patches. *Scale bar 20 μm*. B) MSCs maintained in patches were smaller than those cultured in plates (n = 3 MSC donors). C) Proliferation of MSCs increased after co-culture with rCM in both plate (2D) and collagen patch (3D) but was lower in patches (n = 3 MSC donors). D) Images of CFU-F colonies. CFU-F analysis showed no difference in the number of colonies between MSCs grown in monolayer or in patches, before and after co-culture (n = 3). CFU-F was increased after co-culture by MSCs cultivated in patches (*P* = 0.019) but not in plates (*P* = 0.068). Error bars are SEM. When not specified by a line, * represents the statistical difference within groups (*P <0.05; **P <0.01; ***P <0.001).
(TIF)

S5 Fig Expression of trophic factors by MSCs in plate (2D) and collagen scaffold (3D). MSCs cultured in collagen patches expressed higher levels of BMP4, HGF and VEGF transcripts (n = 4). Error bars are SEM. * represents the statistical difference between groups (**P <0.01; ***P <0.001).
(TIF)

S6 Fig Expression of fibrosis-associated genes by MSCs in 2D and 3D cultures. The expression of fibrosis markers was reduced in MSCs cultured in collagen patches (n = 4 MSC donors). MSCs treated with TGF-β1 were used as positive control. h, human genes; αSMA, alpha-smooth muscle actin; COL I, collagen type I; FN, fibronectin; CTGF, connective tissue growth factor. Error bars are SEM. * represent the statistical significance (*P <0.05; ** *P* <0.01; ***P <0.001).
(TIF)

S7 Fig Expression and activation of TLR3 and TLR4, cytokine/chemokine gene expression by MSCs in plate and collagen scaffold. A) Flow cytometry analysis showed high expression level of TLR3 and TLR4 by MSCs in plates (2D) and collagen patches (3D). B) The activation of NFκB pathway was evaluated by the expression of NFKBIA (NFκB inhibitor alpha). C) Basal expression levels of pro- and anti-inflammatory transcripts were similar in MSCs cultured in plates (2D) and patches (3D), and were upregulated after incubation with Poly(I:C) or LPS (n = 4 MSC donors) (D). Basal expressions are outlined by the dashed line. Error bars are SEM.
(TIF)

S8 Fig Viability of CD4(+) T cells and CD14(+) monocytes in co-culture with MSCs in plate (2D) and collagen scaffold (3D). Respective flow cytometry panels are gated on CD4(+) or CD14(+) cells (n = 3 MSC donors). PI, propidium iodide. Error bars are SEM.
(TIF)
