# Supplementary material for: Collagen scaffold enhances the regenerative properties of mesenchymal stromal cells
Source: PLoS One. 2017 Oct 31;12(10):e0187348. doi: 10.1371/journal.pone.0187348 (PMC5663483; doi:10.1371/journal.pone.0187348)
Supplement: S1 Table — ACTA/αSMA, alpha smooth muscle actin; BMP4, bone morphogenic protein 4; CCL5/ RANTES, C-C motif chemokine ligand 5; COL1A1, collagen type 1 alpha 1; CTGF, connective tissue growth factor; CXCL10/IP10, C-X-C motif chemokine ligand 10; GAPDH, glyceraldehyde 3-phosphate dehydrogenase; HGF, hepatocyte growth factor; IDO1, indoleamine 2,3-dioxygenase 1; IL, interleukin; LIF, leukemia inhibitory factor; NFKBIA, nuclear factor kappa B inhibitor alpha; PDGFA, platelet derived growth factor subunit A; PTGS2/COX2, prostaglandin-endoperoxide synthase 2; TNFAIP6/TSG6, tumor necrosis factor-inducible gene 6 protein; VEGF, vascular endothelial growth factor; * rat-specific sequences. (DOCX) [file pone.0187348.s001.docx]

Table S1. Primers used for real-time PCR

| **Gene** | **Genebank** | **Forward primer** | **Reverse Primer** |  |
| --- | --- | --- | --- | --- |
| ACTA2 | NM_001141945.2 | TTTGGCTTGGCTTGTCAGGG | GGAAGCTTTAGGGTCGCTGG | |
| BMP4 | NM_001202.3 | CGTCCAAGCTATCTCGAGCC | GAATGGCTCCATAGGTCCCTG | |
| CCL5 | NM_001278736.1 | GTCGTCCACAGGTCAAGGAT | GACAAAGCTTGCCCTTGTTCAG | |
| COL1A1 | NM_000088.3 | GCTCTTGCAACATCTCCCCT | CCTTCCTGACTCTCCTCCGA | |
| CTGF | NM_001901.2 | TGTGGCTTTAGGAGCAGTGG | GCTACAGGCAGGTCAGTGAG | |
| CXCL10 | NM_001565.3 | AGCAGAGGAACCTCCAGTCT | ATGCAGGTACAGCGTACAGT | |
| FN1 | NM_001306130.1 | TTACCGTGGGCAACTCTGTC | GTGTAGGGGTCAAAGCACGA | |
| GAPDH | NM_001256799.2 | AATGGGCAGCCGTTAGGAAA | GCCCAATACGACCAAATCAGAG | |
| HGF | NM_000601.4 | CAATGCCTCTGGTTCCCCTT | AGCTCGAAGGCAAAAAGCTG | |
| IDO1 | NM_002164.5 | GCCCTTCAAGTGTTTCACCAA | CCAGCCAGACAAATATATGCGA | |
| IL6 | NM_000600.3 | AAGCCAGAGCTGTGCAGATG | TGGCATTTGTGGTTGGGTCA | |
| IL8 | NM_000584.3 | ACCACCGGAAGGAACCATCT | ACTCCTTGGCAAAACTGCAC | |
| IL10 | NM_000572.2 | TACGGCGCTGTCATCGATTT | TAGAGTCGCCACCCTGATGT | |
| LIF | NM_001257135.1 | TGAAAACTGCCGGCATCTGA | CTGTGTACTGCCGCCAAGA | |
| NFKBIA | NM_020529.2 | GTCAAGGAGCTGCAGGAGAT | TCATGGATGATGGCCAAGT | |
| PDGFA | NM_002607.5 | AGCAGCCAACCAGATGTGAG | AGACCGCACACTGGCAATAA | |
| PTGS2 | NM_000963.3 | GTTCCACCCGCAGTACAGAA | AGGGCTTCAGCATAAAGCGT | |
| TNFAIP6 | NM_007115.3 | AGCACGGTCTGGCAAATACA | ATCCATCCAGCAGCACAGAC | |
| VEGF | NM_001025366.2 | CGCTCGGTGCTGGAATTTG | GTGGGGAATGGCAAGCAAAA | |
| Acta2* | NM_031004.2 | CTGCTCCAGCTATGTGTGAAGA | TTCCAACCATCACTCCCTGG | |
| Col1a1* | NM_053304.1 | AGCTGCATACACAATGGCCTA | TCTTCTTTGCATAGCACGCC | |
| Ctgf* | NM_022266.2 | TAGCAAGAGCTGGGTGTGTG | CAAGGTTCTGACTCCCGACC | |
| Fn* | XM_006245154.2 | AAACCGGGAAGAGCAAGAGG | CCGTTGTCAAAACAGCCAGG | |
| Gapdh* | NM_017008.4 | CTCAGTTGCTGAGGAGTCCC | CCCCTCCTGTTGTTATGGGG | |
| ACTA/αSMA, alpha smooth muscle actin; BMP4, bone morphogenic protein 4; CCL5/ RANTES, C-C motif chemokine ligand 5; COL1A1, collagen type 1 alpha 1; CTGF, connective tissue growth factor; CXCL10/IP10, C-X-C motif chemokine ligand 10; GAPDH, glyceraldehyde 3-phosphate dehydrogenase; HGF, hepatocyte growth factor; IDO1, indoleamine 2,3-dioxygenase 1; IL, interleukin; LIF, leukemia inhibitory factor; NFKBIA, nuclear factor kappa B inhibitor alpha; PDGFA, platelet derived growth factor subunit A; PTGS2/COX2, prostaglandin-endoperoxide synthase 2; TNFAIP6/TSG6, tumor necrosis factor-inducible gene 6 protein; VEGF, vascular endothelial growth factor; * rat-specific sequences. | | | | |
