# Supplementary figures and images for: Collagen scaffold enhances the regenerative properties of mesenchymal stromal cells
Source: PLoS One. 2017 Oct 31;12(10):e0187348. doi: 10.1371/journal.pone.0187348 (PMC5663483; doi:10.1371/journal.pone.0187348)

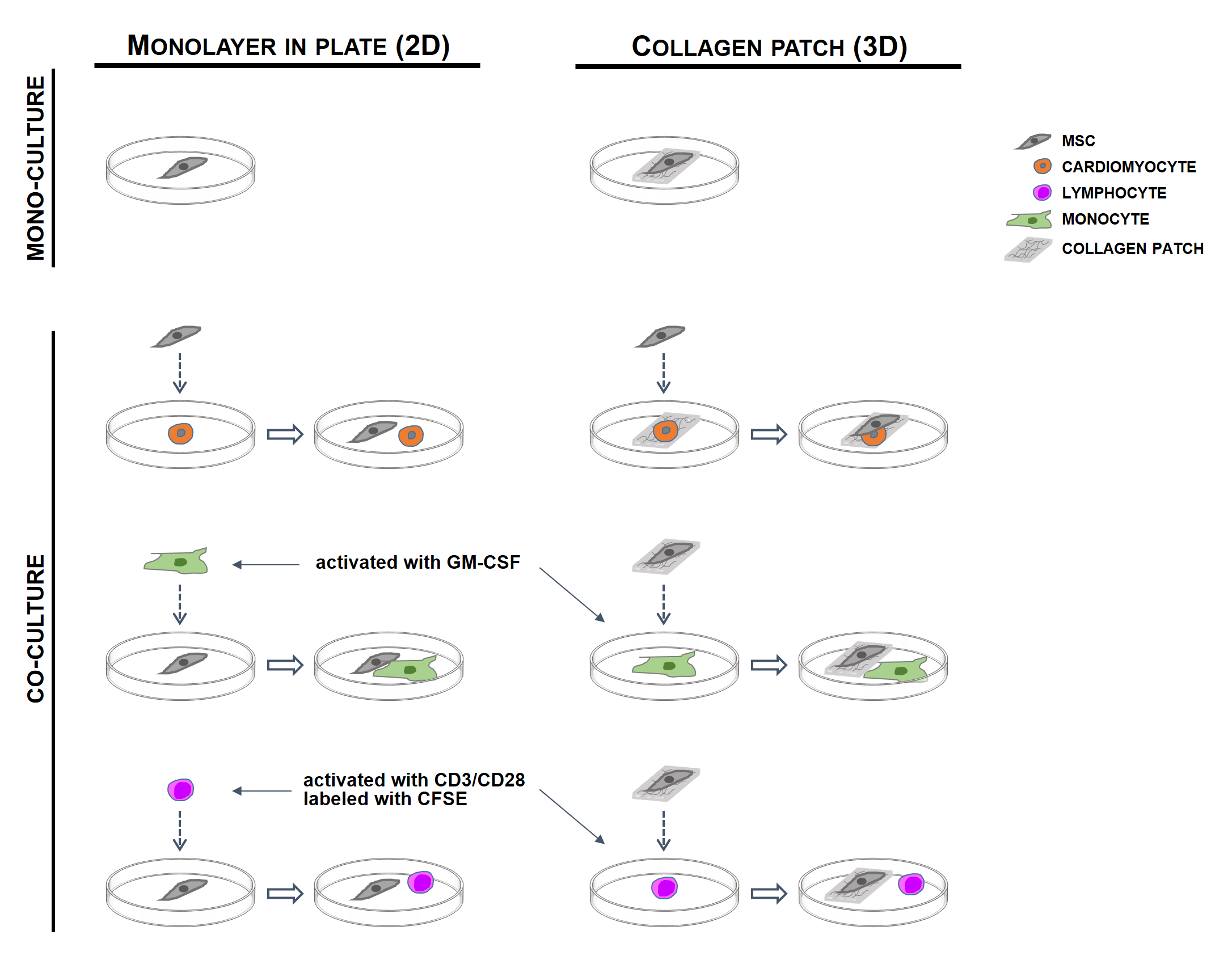

Supplement: S1 Fig — MSCs were either mono- or co-cultured with other cells in plates or collagen patches. Rat cardiac cell extracts consist of cardiomyocyte and cardiac fibroblast (only cardiomycote is depicted in the figure). If applicable. MSCs underwent treatment before being washed and used in subsequent experiments. Monocytes were activated with GM-CSF (granulocyte macrophage colony stimulating factor) and lymphocytes were activated with CD3/CD28 beads and stained with CFSE (carboxyfluorescein succinimidyl ester) before co-culture with MSCs. Single cell suspensions were prepared by trypsinizing the cells in plates or digesting patches with collagenase. (TIF) [file pone.0187348.s002.tif]

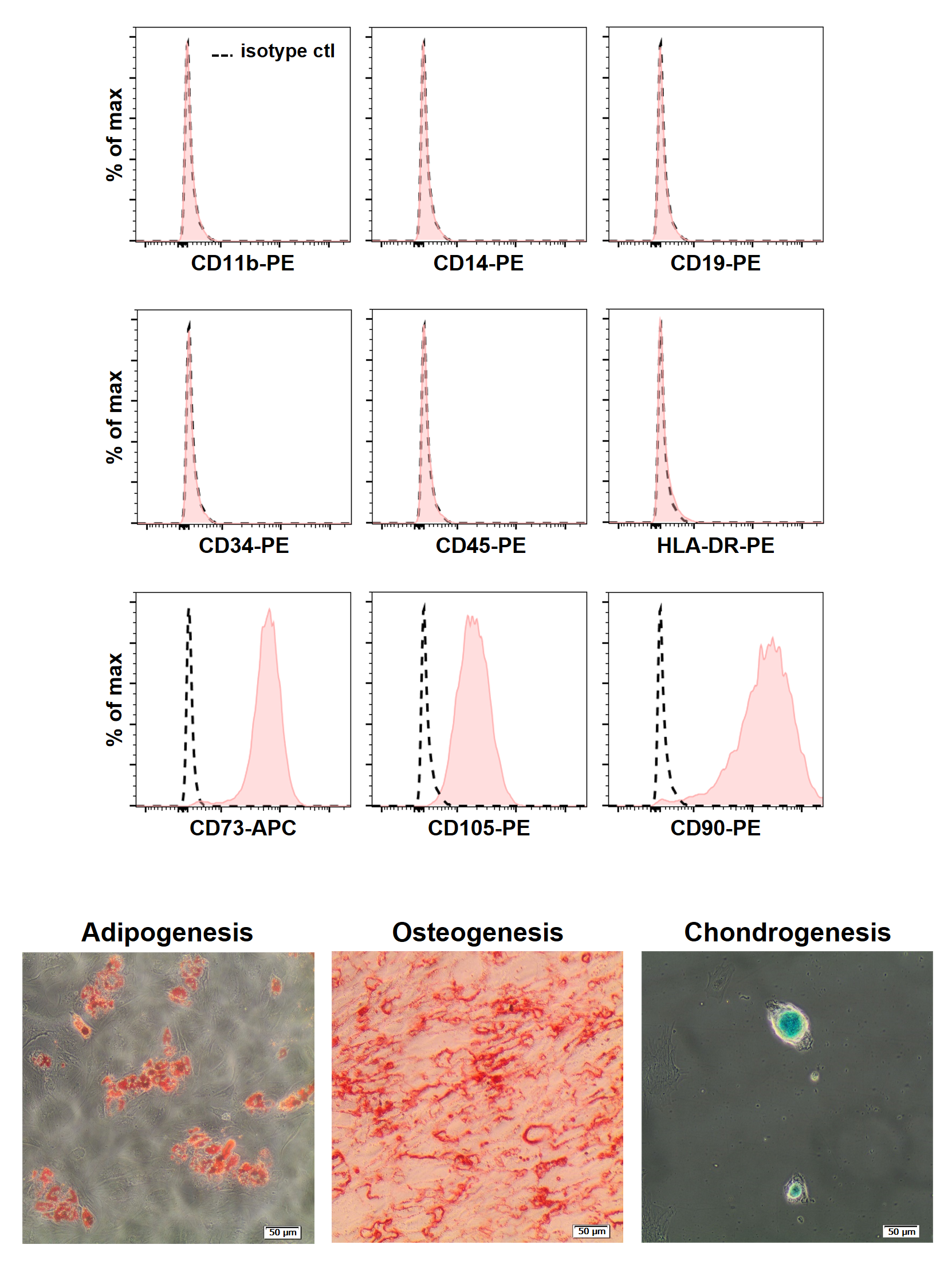

Supplement: S2 Fig — A) Flow cytometry analysis of MSCs showing the expression of CD73, CD105, CD90 and lack of the expression of hematopoietic markers CD11b, CD14, CD19, CD34, CD45, and HLA-DR2 by MSCs. Dashed lines are isotype controls. B) Tri-lineage differentiation of MSCs showing adipogenic (Oil Red O staining), osteogenic (Alizarin Red staining) and chondrogenic (Alician Blue staining). Scale bar 50 μm. (TIF) [file pone.0187348.s003.tif]

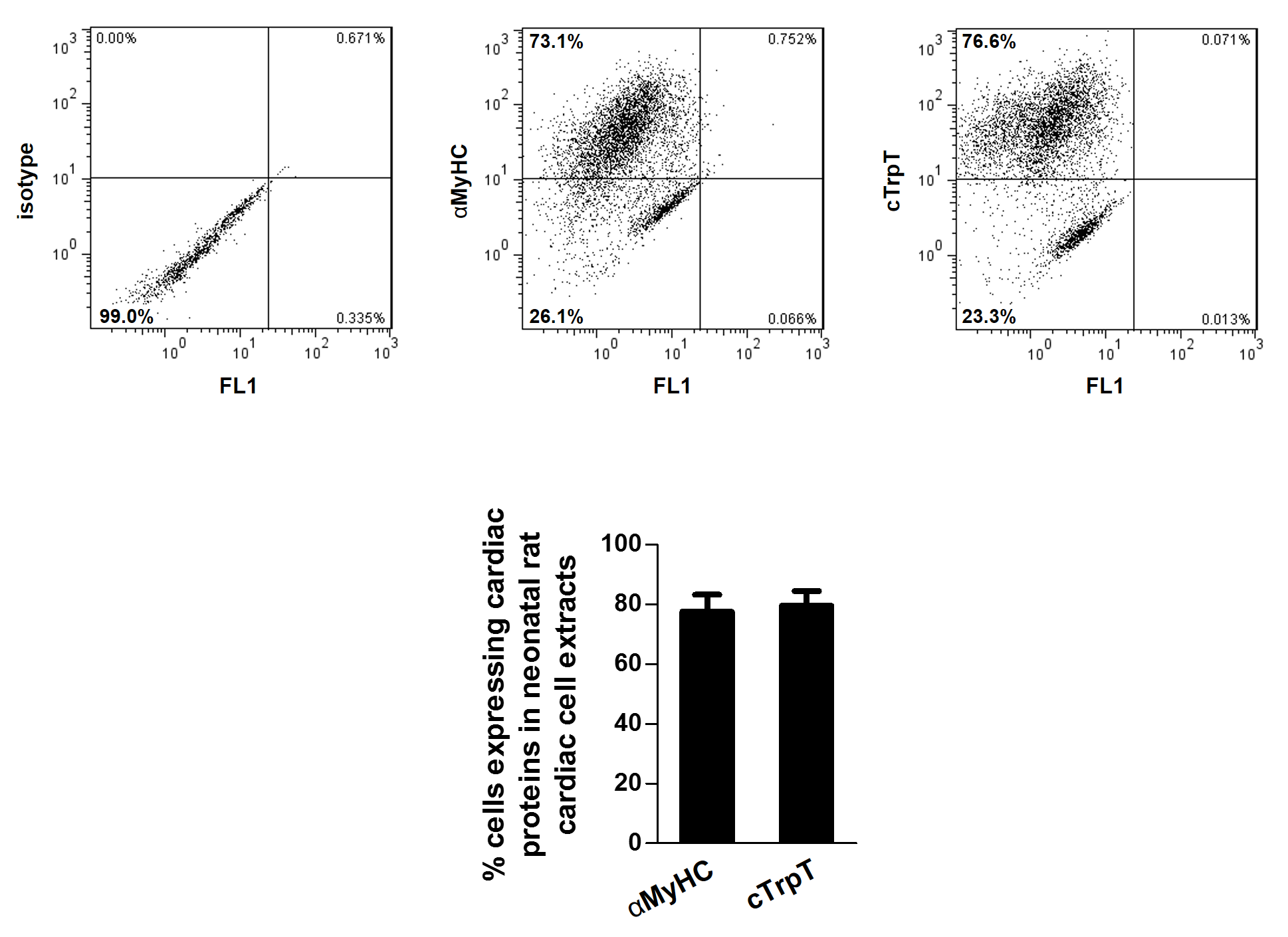

Supplement: S3 Fig — Rat cardiac cell extracts mostly contain cardiomyocytes expressing α-myosin heavy chain (αMyHC) and cardiac troponin T (cTrpT) proteins. Cardiac fibroblasts stain negative for cardiac markers. n = 3 independent isolations. Error bars are SEM. (TIF) [file pone.0187348.s004.tif]

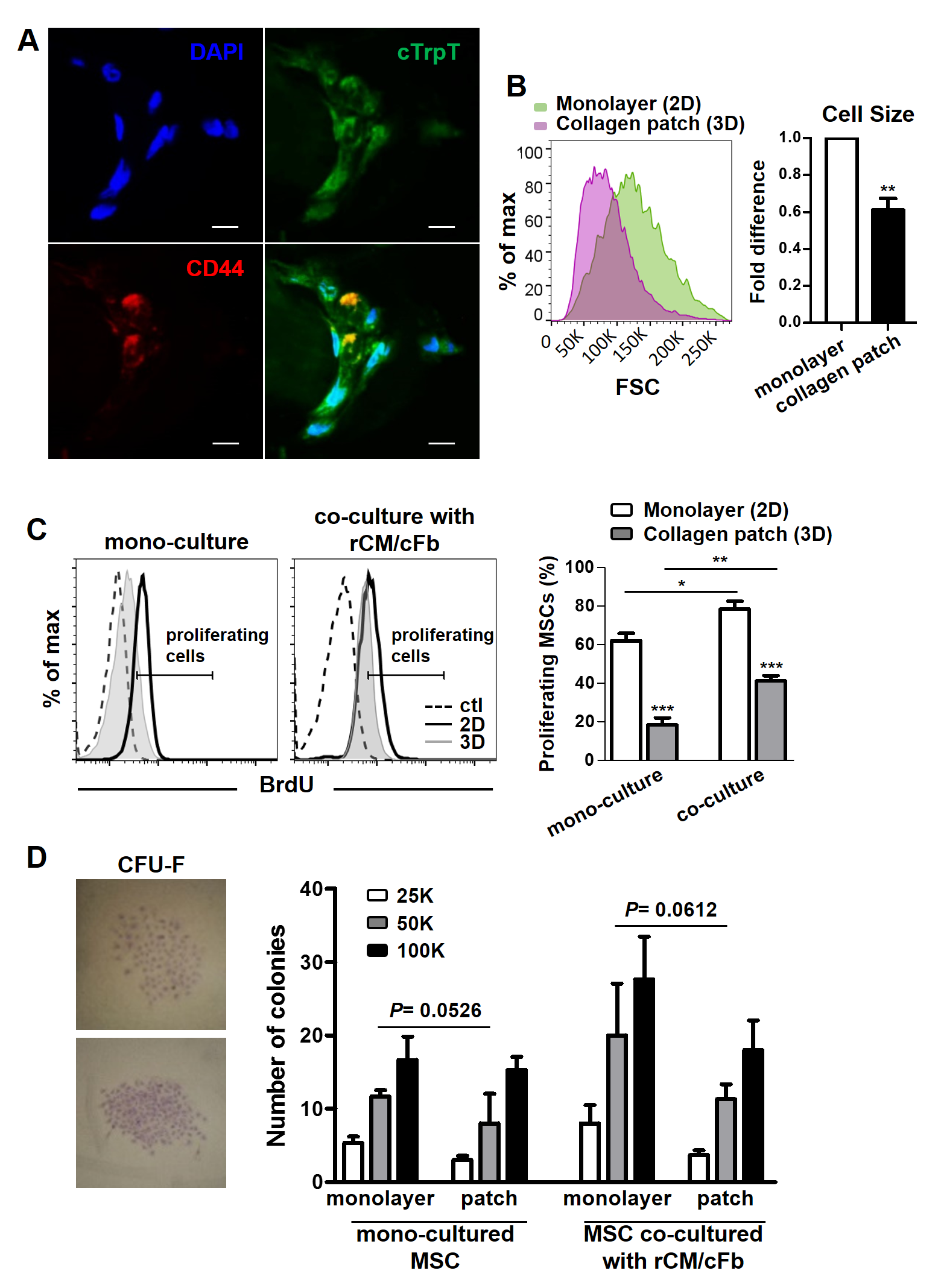

Supplement: S4 Fig — A) Immunohistochemistry staining showing the expression of cTrpT by MSCs co-cultured with neonatal rat cardiomyocyte/cardiac fibroblasts (rCM/cFb) in collagen patches. Scale bar 20 μm. B) MSCs maintained in patches were smaller than those cultured in plates (n = 3 MSC donors). C) Proliferation of MSCs increased after co-culture with rCM in both plate (2D) and collagen patch (3D) but was lower in patches (n = 3 MSC donors). D) Images of CFU-F colonies. CFU-F analysis showed no difference in the number of colonies between MSCs grown in monolayer or in patches, before and after co-culture (n = 3). CFU-F was increased after co-culture by MSCs cultivated in patches (P = 0.019) but not in plates (P = 0.068). Error bars are SEM. When not specified by a line, * represents the statistical difference within groups (*P <0.05; **P <0.01; ***P <0.001). (TIF) [file pone.0187348.s005.tif]

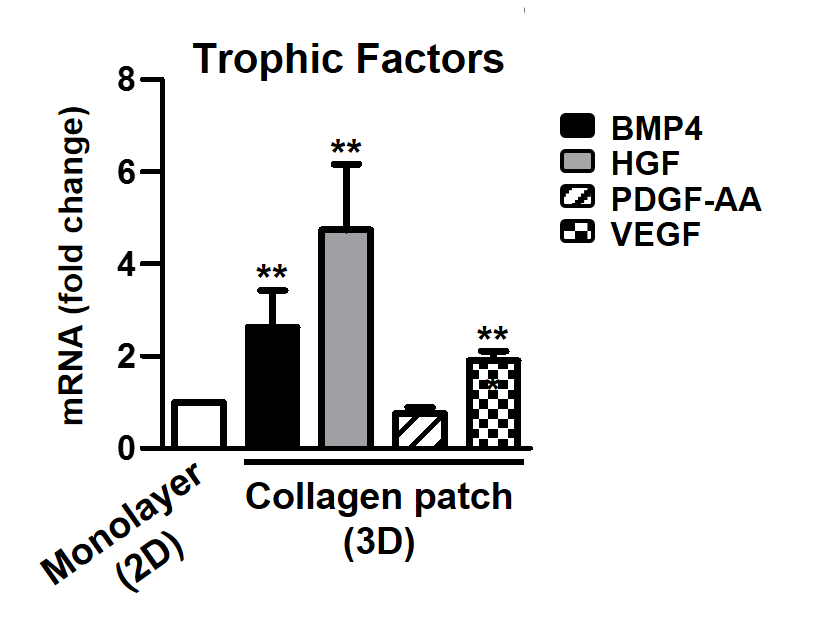

Supplement: S5 Fig — MSCs cultured in collagen patches expressed higher levels of BMP4, HGF and VEGF transcripts (n = 4). Error bars are SEM. * represents the statistical difference between groups (**P <0.01; ***P <0.001). (TIF) [file pone.0187348.s006.tif]

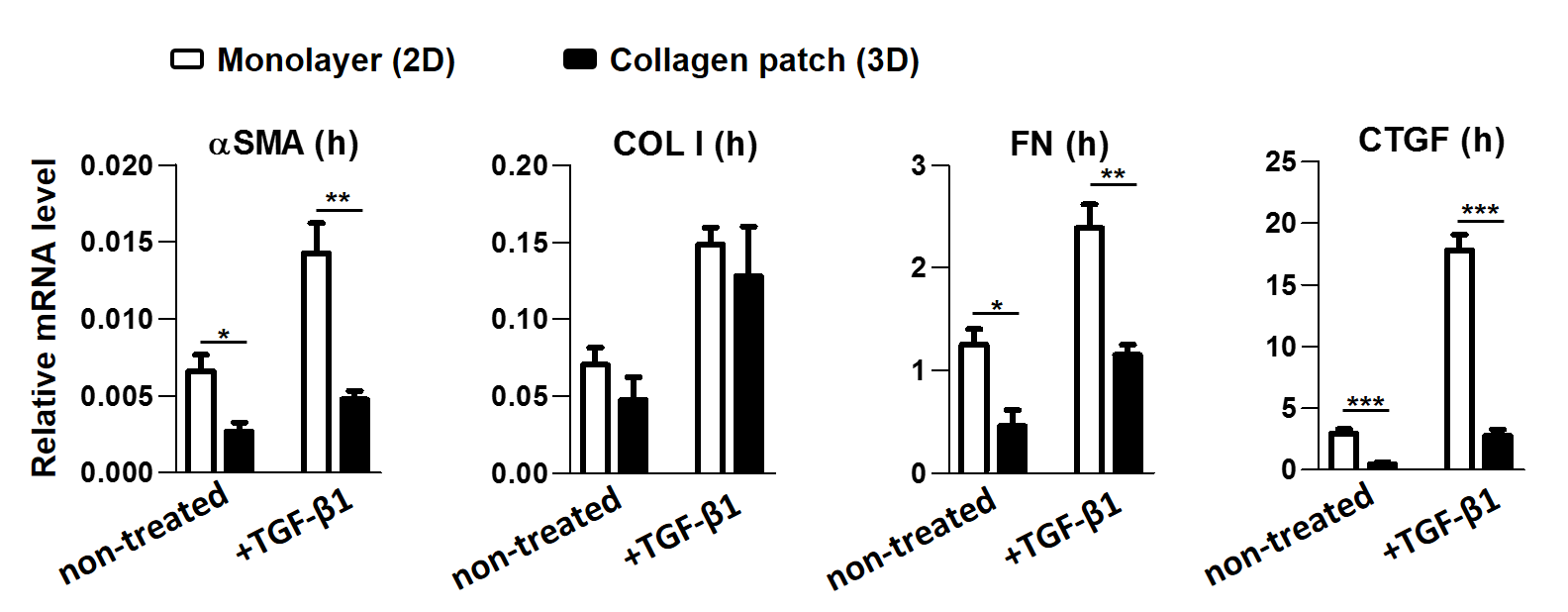

Supplement: S6 Fig — The expression of fibrosis markers was reduced in MSCs cultured in collagen patches (n = 4 MSC donors). MSCs treated with TGF-β1 were used as positive control. h, human genes; αSMA, alpha-smooth muscle actin; COL I, collagen type I; FN, fibronectin; CTGF, connective tissue growth factor. Error bars are SEM. * represent the statistical significance (*P <0.05; ** P <0.01; ***P <0.001). (TIF) [file pone.0187348.s007.tif]

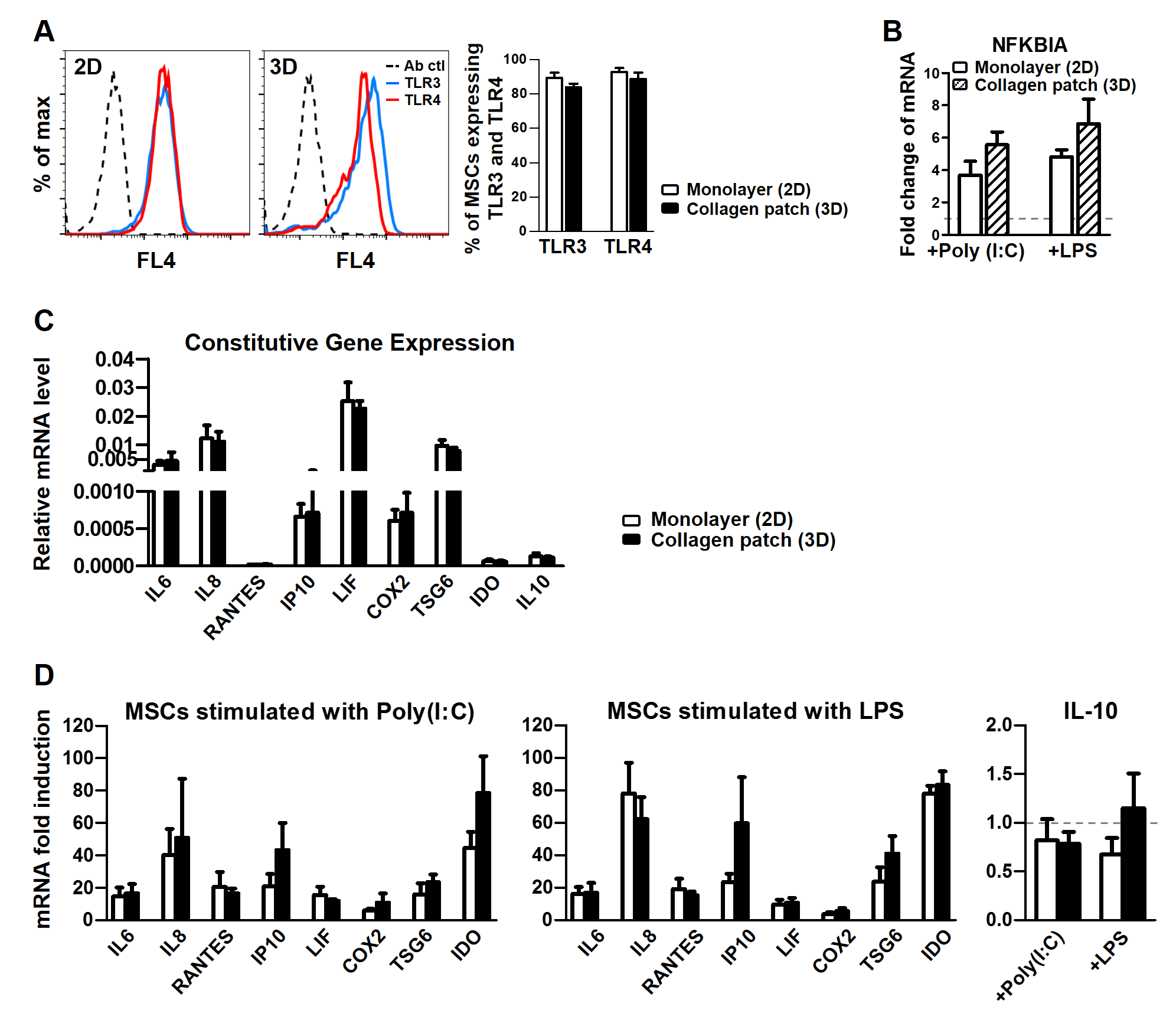

Supplement: S7 Fig — A) Flow cytometry analysis showed high expression level of TLR3 and TLR4 by MSCs in plates (2D) and collagen patches (3D). B) The activation of NFκB pathway was evaluated by the expression of NFKBIA (NFκB inhibitor alpha). C) Basal expression levels of pro- and anti-inflammatory transcripts were similar in MSCs cultured in plates (2D) and patches (3D), and were upregulated after incubation with Poly(I:C) or LPS (n = 4 MSC donors) (D). Basal expressions are outlined by the dashed line. Error bars are SEM. (TIF) [file pone.0187348.s008.tif]

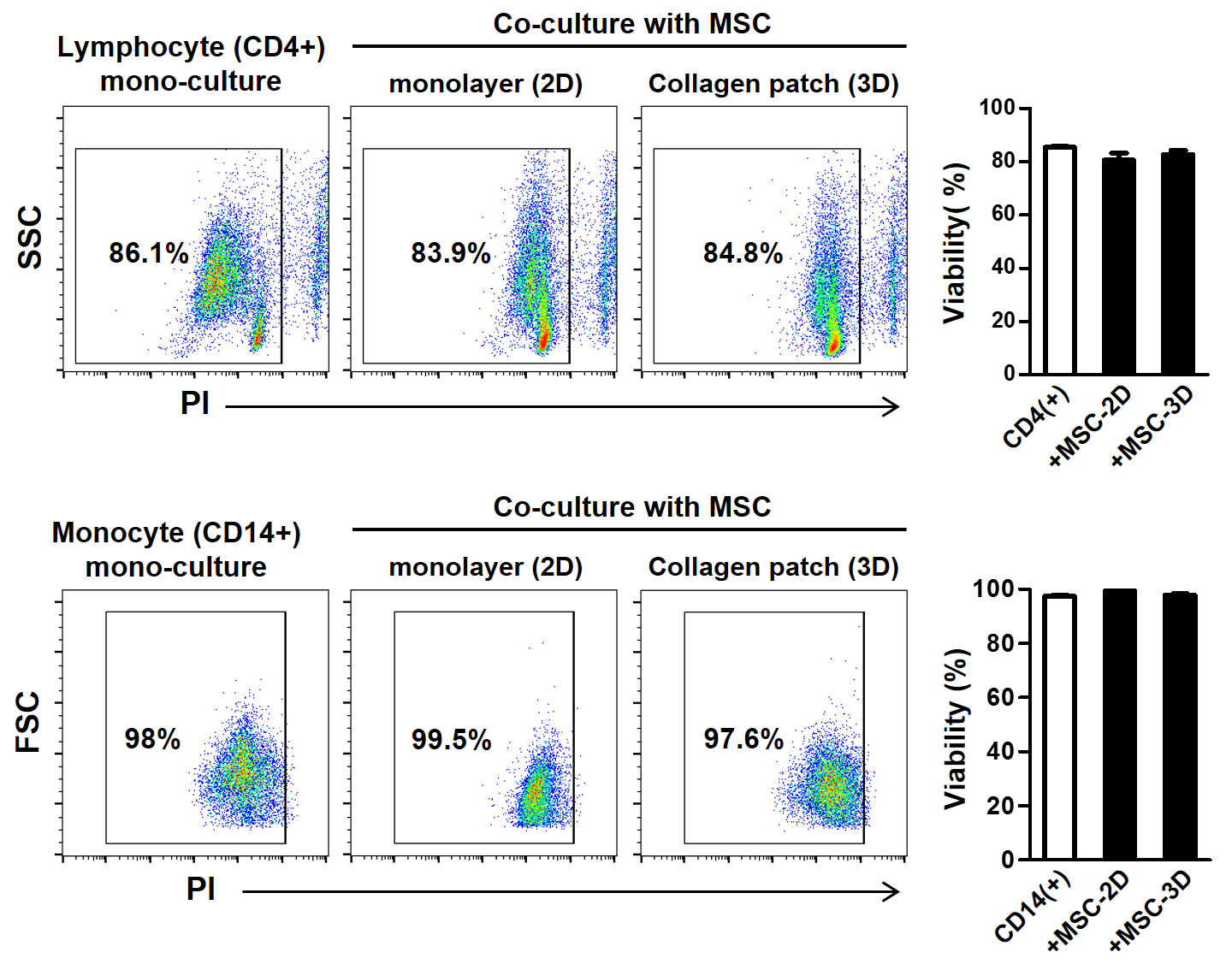

Supplement: S8 Fig — Respective flow cytometry panels are gated on CD4(+) or CD14(+) cells (n = 3 MSC donors). PI, propidium iodide. Error bars are SEM. (TIF) [file pone.0187348.s009.tif]
